# Proteomic analysis distinguishes extracellular vesicles produced by cancerous versus healthy pancreatic organoids

**DOI:** 10.1038/s41598-022-07451-6

**Published:** 2022-03-03

**Authors:** Abigail C. Buenafe, Craig Dorrell, Ashok P. Reddy, John Klimek, Daniel L. Marks

**Affiliations:** 1 Papé Family Pediatric Research Institute, Oregon Health and Science University, Portland, OR USA; 2 Oregon Stem Cell Center, Papé Family Pediatric Research Institute, Oregon Health and Science University, Portland, OR USA; 3 Proteomics Shared Resource, Oregon Health and Science University, Portland, OR USA; 4 Knight Cancer Institute, Oregon Health and Science University, Portland, OR USA; 5 Brenden-Colson Center for Pancreatic Care, Oregon Health and Science University, Portland, OR USA

**Keywords:** Biological techniques, Cancer, Biomarkers

## Abstract

Extracellular vesicles (EVs) are produced and released by both healthy and malignant cells and bear markers indicative of ongoing biological processes. In the present study we utilized high resolution flow cytometry to detect EVs in the plasma of patients with pancreatic ductal adenocarcinoma (PDAC) and in the supernatants of PDAC and healthy control (HC) pancreatic organoid cultures. Using ultrafiltration and size exclusion chromatography, PDAC and HC pancreatic organoid EVs were isolated for mass spectrometry analysis. Proteomic and functional protein network analysis showed a striking distinction in that EV proteins profiled in pancreatic cancer organoids were involved in vesicular transport and tumorigenesis while EV proteins in healthy organoids were involved in cellular homeostasis. Thus, the most abundant proteins identified in either case represented non-overlapping cellular programs. Tumor-promoting candidates LAMA5, SDCBP and TENA were consistently upregulated in PDAC EVs. Validation of specific markers for PDAC EVs versus healthy pancreatic EVs will provide the biomarkers and enhanced sensitivity necessary to monitor early disease or disease progression, with or without treatment. Moreover, disease-associated changes in EV protein profiles provide an opportunity to investigate alterations in cellular programming with disease progression.

## Introduction

Extracellular vesicles (EVs) recently received wide attention as mediators of intercellular communication and regulators of diverse biological processes^1,2^. Analyzed EVs include both exosomes, which are 30–150 nm diameter vesicles generated as endosomal-derived multivesicular bodies (MVB), and 100 to > 500 nm microvesicles derived from the plasma membrane. Both vesicle types are known to carry functional biomolecules including protein, nucleic acid and lipid as cargo. Significantly, EVs are functionally involved in many cancer-supportive processes^3^. As reviewed elsewhere, EVs have been found to play a role in promoting tumor growth by conditioning of the primary as well as secondary tumor sites^4–6^, by mediating immune suppression^7,8^ and by mediating mechanisms of drug resistance^9–12^.

In pancreatic cancer studies, EVs are being investigated for functional roles in communication within the tumor microenvironment and in metastasis^13,14^. Due to the fact that overt symptoms arise only at later stages of the disease, a diagnosis of pancreatic cancer is typically associated with a devastating prognosis. Thus, efforts to detect pancreatic cancer markers at earlier disease stages now include the investigation of EVs not only produced by the tumor cells themselves^15^, but also by the cellular microenvironment influenced by the tumor^16–19^. Thus far, the identification of early disease EV-associated cancer biomarkers is limited, perhaps due to the fact that most markers are expressed to some extent by non-cancerous cells and the protocols used to isolate and identify disease-relevant EVs are still being developed^20^. EV-associated pancreatic cancer biomarkers currently being studied for diagnostic purposes include surface markers such as GPC1^21,22^ and ANXA6^17^, as well as EV cargo such as mutant KRAS^+^ DNA^23,24^ and miRNA^25–27^. Screening with a panel of EV biomarkers would significantly improve the sensitivity and specificity of detecting pancreatic cancer at an early stage^28^.

With the multitude of diverse roles ascribed to EVs, it is important to recognize that there remain significant challenges to the isolation and characterization of defined EV populations^29^. Analysis of any biofluid sample will present a host of non-EV particles in the same nano-size range, such as lipoproteins and protein aggregates, much of which will co-purify with EVs^30–32^. An additional challenge with EV-containing biofluids is that most isolation and characterization methods rely on bulk measurements of a heterogeneous EV population^33^. This becomes a major issue when disease relevant EVs are largely diluted by the majority of normal EVs present, with the chance of detection earlier in disease thereby being greatly diminished. The International Society for Extracellular Vesicles (ISEV) put forth guidelines^34–36^ for the conductance and reporting of EV research to provide increased validation and comparable results between different research studies. The concepts presented in those guides are supported in the work reported here.

Three-dimensional organoid cultures are derived from primary tissues, are capable of self-renewal and retain functional and genetic characteristics of the original tissue. Organoids have been established from both normal and cancerous tissue, including pancreatic cancer^37^, providing a powerful tool for the analyses of patient-derived samples. In this current study, we utilized a combination of ultrafiltration and size exclusion chromatography (SEC) to characterize the proteomic profiles of EVs isolated from pancreatic ductal adenocarcinoma (PDAC) and healthy control (HC) pancreatic organoid cultures. Altogether our results present a rational method for the identification of EV protein biomarkers of pancreatic cancer. The ability to multiplex tissue-specific EV biomarkers together with validated cancer-associated EV biomarkers should lead to an enhanced screening method utilizing EV biomarker profiles specific for pancreatic or other types of cancers.

## Methods

### Antibodies and reagents

Mouse monoclonal antibodies to human surface markers were obtained from BD Biosciences (BD Biosciences, San Jose, CA, USA): CD9-PE (555372), CD45-PE (555483), CD63-AF647 (561983), CD41a-APC-H7 (561422). CD45-APC (304012) was obtained from BioLegend (San Diego, CA, USA). Tspan8-AF647 (FAB4734R) was obtained from R&D Systems (Minneapolis, MN, USA). CD81-PE (A15781) antibody was obtained from ThermoFisher/Invitrogen. Megamix beads were a gift from BD Biosciences and are available from BioCytex/Stago (Marseille, France). Fluoresbrite YG Microsphere beads (fl-200 nm beads) were obtained from Polysciences Inc. (Warrington, PA, USA).

### Processing of plasma samples

Human plasma samples were obtained through the Brenden-Colson Center for Pancreatic Care (BCCPC) in accordance with regulations approved by the Institutional Review Board at Oregon Health and Science University (Oregon Pancreas Tissue Registry, protocol #3609). Experimental procedures were approved and performed in accordance with all regulations. Informed consent was obtained from all subjects. A detailed summary of patient plasma samples used for EV analysis is provided in Supplementary Table S1. Blood samples were collected into vacutainer tubes containing EDTA to prevent coagulation and all samples were processed within two hours of the draw. Samples were centrifuged twice at 2500×*g* for 15 min at room temperature to obtain platelet-poor plasma (PPP) and supernatant was transferred into clean tubes after each spin, taking care to avoid contamination from the blood cell pellet. PPP was then aliquoted and stored at − 80 °C. Samples were thawed only once before analysis. Unless specified otherwise, all plasma samples in this study were stained and analyzed by flow cytometry within one week of processing.

As an additional control, EV marker stability over time in storage was evaluated. On the day of collection, samples were processed, then aliquoted and stored at − 80 °C until analysis. Plasma samples were thawed only once and analyzed for CD9 at two different timepoints separated by a period of eight to eleven weeks. As shown in Supplemental Fig. S1, a lower frequency of CD9+ events was detected at the second time point, with the percent decrease varying between samples. Additional investigation is needed to determine what contributes to EV marker instability during storage or why some samples are more susceptible than others. Whether this is an issue related to a particular processing or storage step remains to be determined. While EV markers may still be detectable months or perhaps even years after sample collection, any quantitative or comparative measurement of EV marker levels should be performed as soon as possible after sample collection.

### Pancreatic organoid cultures

Tissue source and cell isolation: human PDAC tumor specimens were obtained in accordance with protocol #3609 approved by the Institutional Review Board at Oregon Health and Science University. Experimental procedures were approved and performed in accordance with all regulations. A detailed summary of patient pancreatic tumor samples used to generate organoid cultures for EV analysis is provided in Supplementary Table S2. Normal healthy pancreatic organoids were established in an identical manner from islet preparations obtained through the Integrated Islet Distribution Program (https://iidp.coh.org). Cells were obtained by sequential digestion with collagenase and trypsin^38^; a portion of these cells was used to initiate organoid cultures and the remainder were cryopreserved for future culture and analysis.

Organoid culture: Pancreatic organoids were initiated, cultured and cryopreserved as described previously^39^ with human growth factors used instead of mouse factors in the liquid media. Supplementary Table S3 provides a list of all media components used in the generation of human pancreatic organoids. A total of 10^5^ cells were embedded per 50 µl Matrigel droplet in a 24-well plate, with 500 µl of defined media added once the droplets had hardened. Cultures were maintained with biweekly media changes until the droplet was packed with organoids, after which the droplet and organoids were dispersed to fragments by trypsinization and re-embedded in a new Matrigel droplet. For harvest of EVs, cryopreserved patient-specific organoids from passage 8–12 were thawed and cultured starting with four multiple 50 µl droplets. Supernatant was collected and replaced with fresh media every 2 days and stored at − 80 °C after sequential centrifugation at 300×*g* for 10 min and at 2000×*g* for 10 min, both at room temperature, to clarify supernatants. Media replacement and culture passage was continued until 20–30 ml of frozen supernatant was accumulated for further processing. One culture passage, in which each droplet was trypsinized and re-embedded in two daughter droplets, was required to prevent organoid overgrowth. Media was discarded and replaced 48 h after this passage—to minimize any passage-related changes in EV characteristics—then collection was resumed. Phase contrast images of live organoid cultures were photographed using an EVOS XL microscope (Echo Inc., San Diego, CA).

### Staining and analysis of EV samples by flow cytometry

Flow cytometry analysis of plasma and culture supernatants was performed on a Becton–Dickinson FACSCanto II (BD, San Jose, CA) modified with a variable output blue (488-nm) laser. All measurements reported here were performed using the 200 mW setting. Voltage settings for forward scatter and side scatter were set at 475 and 450, respectively. A 0.1 µm filter was installed to reduce background counts contributed by the sheath fluid (1 × PBS). Any dilution of samples pre- or post-staining was made using 0.1 µm-filtered PBS. The threshold on the FACSCanto II was set at 300 using the side scatter parameter, and side scatter was plotted against fluorescence for EV marker detection. Gates were set to count fluorescent positive particles within an established size range (< 300 nm) excluding any antibody debris. Other relevant specifications for this FACSCanto II included: Nozzle size (180 µm), sheath pressure (4.3 psi), high flow rate (120 µl/min), low flow rate (10 µl/min). Details for flow cytometry procedure, instrument settings, and reagents are organized in Supplementary Tables S4–S6 according to the Minimal Information for Flow Cytometry of EV-specific reporting (MIFlowCyt-EV) framework^40^.

Staining of plasma EV samples: 10 µl of PPP sample was incubated with 1 µl of labeled monoclonal antibody at the predetermined optimal concentration for 2 h at room temperature. After the staining period, 10 µl of freshly diluted fl-200 nm bead solution was added to the stained EVs, and samples were diluted into 0.1 µm-filtered PBS for flow analysis. Dilution varied by sample and was adjusted such that less than 10K events/s would be collected at the lowest flow rate to prevent coincident detection^41,42^. At this event rate, the abort rate was < 10% and further dilution confirmed that the event rate decreased proportionally. Collection of event data was started after 30 s of run time to ensure equilibration of sample flow rate. The stopping gate was set such that events were collected until the 200 nm bead count was equal to 1000 in the 488-1 channel. Replicates were set up and stained as independent samples in separate tubes. To verify the presence of antibody-stained vesicles, samples were treated with 0.1% Triton X-100 and then flow analyzed again to verify the loss of signal due to vesicle disruption^41,43^. Enumeration of nanoparticles within the range analyzed (< 300 nm relative to bead size) and of fluorescently labeled EVs was performed using FlowJo analysis software version 10.1r7 (FlowJo LLC, Ashland, OR, USA).

Staining of culture supernatants or column fractions: 10 µl of clarified supernatant or SEC column fraction (see EV isolation below) was incubated with 1 µl of labeled monoclonal antibody for 2 h at room temperature. SEC fractions were stained with a suitable tetraspanin marker as determined from previously screened supernatants. Stained samples were diluted 25-fold and events were collected for 3 min under medium flow rate.

### EV isolation procedures

100 µl of PPP was applied to a qEV mini SEC column (Izon Science, Medford, MA, USA) and fractions collected according to manufacturer’s recommendation. Briefly, upon sample application, 1 ml void volume was collected followed by 5 fractions of 200 µl each. All fractions were assessed for EV binding of labeled antibody by flow cytometry as described above, and for protein measurement of OD at 280 nm on a Nanodrop 2000 (ThermoFisher Scientific). EV size distribution and concentration were assessed by Tunable Resistive Pulse Sensing using a qNano Gold instrument.

For the isolation of EVs prior to mass spectrometry analysis, organoid culture supernatant was collected and screened for EV production by flow cytometry. Supernatants were pre-filtered through 0.8 µm Whatman cellulose acetate disc filters (GE Life Sciences, Marlborough, MA, USA) and then concentrated in Amicon-15 CA filters (100KDa MWCO, Millipore Sigma, Burlington, MA, USA) at 2000×*g* for 15 min per spin until samples were reduced to < 2 ml. Samples were then washed for 3 additional spins with 0.1 µm-filtered PBS. 500 µl of concentrated sample was applied to a qEV original SEC column (Izon Science, Medford, MA, USA) and fractions collected according to manufacturer’s recommendation. The first 3 ml were collected as the void volume followed by the collection of at least six fractions at 0.5 ml each. Void volume and fractions were assessed for EV detection by flow cytometry and by Nanodrop measurement of OD at 280 nm.

### Transmission electron microscopy of EVs

5 µl of SEC-isolated EVs were deposited onto glow discharged (120 s 15 mAmp, negative mode) carbon formvar 400 Mesh copper grids (Ted Pella 01822-F) for 3 min, rinsed for 15 s in water, wicked on Whatman filter paper 1, stained for 3 min in freshly prepared 1% (w/v) uranyl acetate in water, wicked and air dried. Samples were imaged at 120 kV on a FEI Tecnai™ Spirit TEM system. Images were acquired as 2048 × 2048 pixel, 16-bit gray scale files using the FEI’s TEM Imaging and Analysis (TIA) interface on an Eagle™ 2K CCD multiscan camera. Nanoparticle density assessed by flow cytometry in the EV fractions after size exclusion chromatography ranged from 2.4 to 3.6 × 10^5^ events/µl.

### Mass spectrometry

#### Sample preparation

Pancreatic organoid EV samples were pooled from fractions 1 and 2 after SEC isolation then dried in a speedvac. Samples were digested using PreOmics iST-NHS Kit 12 × (Product# PO00026) following the manufacturer’s instructions. Following digestion, all samples were taken to dryness by vacuum centrifugation, dissolved in 50 µl each of HPLC water and peptide concentrations determined using the Pierce Quantitative Colorimetric Peptide Assay Kit (Thermo Scientific). Total peptide recovery for EV samples in the 4 × 4 study was as follows: PDAC-1 (3.9 µg), PDAC-2 (6.3 µg), PDAC-3 (5.7 µg), PDAC-4 (4.3 µg), HC-1 (3.8 µg), HC-2 (1.5 µg), HC-3 (4.4 µg), HC-4 (3.5 µg). Samples PDAC-5 through PDAC-10 were processed separately and total peptide yields ranged from 5 to 20 µg.

#### LC–MS/MS

Sample digests (~ 4 µg, with the exception of HC-2) were loaded onto an Acclaim PepMap 0.1 × 20 mm NanoViper C18 peptide trap (Thermo Scientific) for 5 min at a 10 μl/min flow rate in a 2% acetonitrile, 0.1% formic acid mobile phase and peptides separated using a PepMap RSLC C18, 2 μm particle, 75 μm × 50 cm EasySpray column (Thermo Scientific) using a 7.5–30% acetonitrile gradient over 200 min in mobile phase containing 0.1% formic acid and a 300 nl/min flow rate using a Dionex NCS-3500RS UltiMate RSLC nano UPLC system. Tandem mass spectrometry data was collected using an Orbitrap Fusion mass spectrometer configured with an EasySpray NanoSource (Thermo Scientific). Instrument was configured for data dependent analysis (DDA) using the MS/DD-MS/MS setup. Full MS resolutions were set to 60,000 at m/z 200 and full MS AGC target was 3E6 with an IT of 50 ms. Mass range was set at 370–1400. AGC target value for fragment spectra was set at 1E5, and intensity threshold was kept at 2E5. Isolation width was set at 1.2 m/z. A fixed first mass of 100 m/z was used. Normalized collision energy was set at 28%. Peptide match was set to off, and isotope exclusion was on. All data were acquired in profile mode using positive polarity.

#### Data analysis

Comet (v. 2016.01, rev. 2)^44^ was used to search MS2 Spectra against an April 2020 version of a uniprot FASTA protein database containing canonical Homo sapiens sequences, concatenated sequence-reversed entries to estimate error thresholds and 179 common contaminant sequences and their reversed forms. The database processing was performed with python scripts available at https://github.com/pwilmart/fasta_utilities.git and Comet results processing used the PAW pipeline^45^ from https://github.com/pwilmart/PAW_pipeline.git. Comet searches for all samples were performed with trypsin enzyme specificity. Monoisotopic parent ion mass tolerance was 1.25 Da. Monoisotopic fragment ion mass tolerance was 1.0005 Da. A static modification of + 113.084 Da was added to all cysteine residues and a variable modification of + 15.9949 Da to methionine residues. We used a linear discriminant transformation to improve the identification sensitivity from the Comet analysis^45,46^. Comet scores combined into linear discriminant function scores, and discriminant score histograms created separately for each peptide charge state (2+, 3+, and 4+). Separate histograms were created for matches to forward sequences and for matches to reversed sequences for all peptides of 7 amino acids or longer. The score histograms for reversed matches were used to estimate peptide false discovery rates (FDR) and set score thresholds for each peptide class. For the 4 × 4 study, a total of 781 proteins were identified across all 8 samples (4 PDAC and 4 HC) after removing contaminants (e.g., keratin, trypsin) and reversed entries. The total number of proteins identified for each sample is as follows: PDAC-1 (548), PDAC-2 (534), PDAC-3 (507), PDAC-4 (595), HC-1 (573), HC-2 (510), HC-3 (556), HC-4 (576). Differential protein abundance between groups was determined by comparing the spectral counts of identified proteins between experimental groups using the Bioconductor package edgeR^47^. Comparisons between groups were performed using Fisher's exact test, and a Benjamini–Hochberg multiple testing correction used to limit the candidate false discovery rate. The mass spectrometry proteomics data have been deposited to the ProteomeXchange Consortium via the PRIDE^48^ partner repository with the dataset identifier PXD030325.

### Ethics approval and consent to participate

All PDAC samples, including blood and tumor tissue, were collected in accordance with regulations approved by the Institutional Review Board at Oregon Health and Science University (Oregon Pancreas Tissue Registry, protocol #3609). Experimental procedures were approved and performed in accordance with all regulations. Informed consent was obtained from all subjects.

## Results

### Detection of EV surface markers in human PDAC plasma by flow cytometry

**Figure 1:**
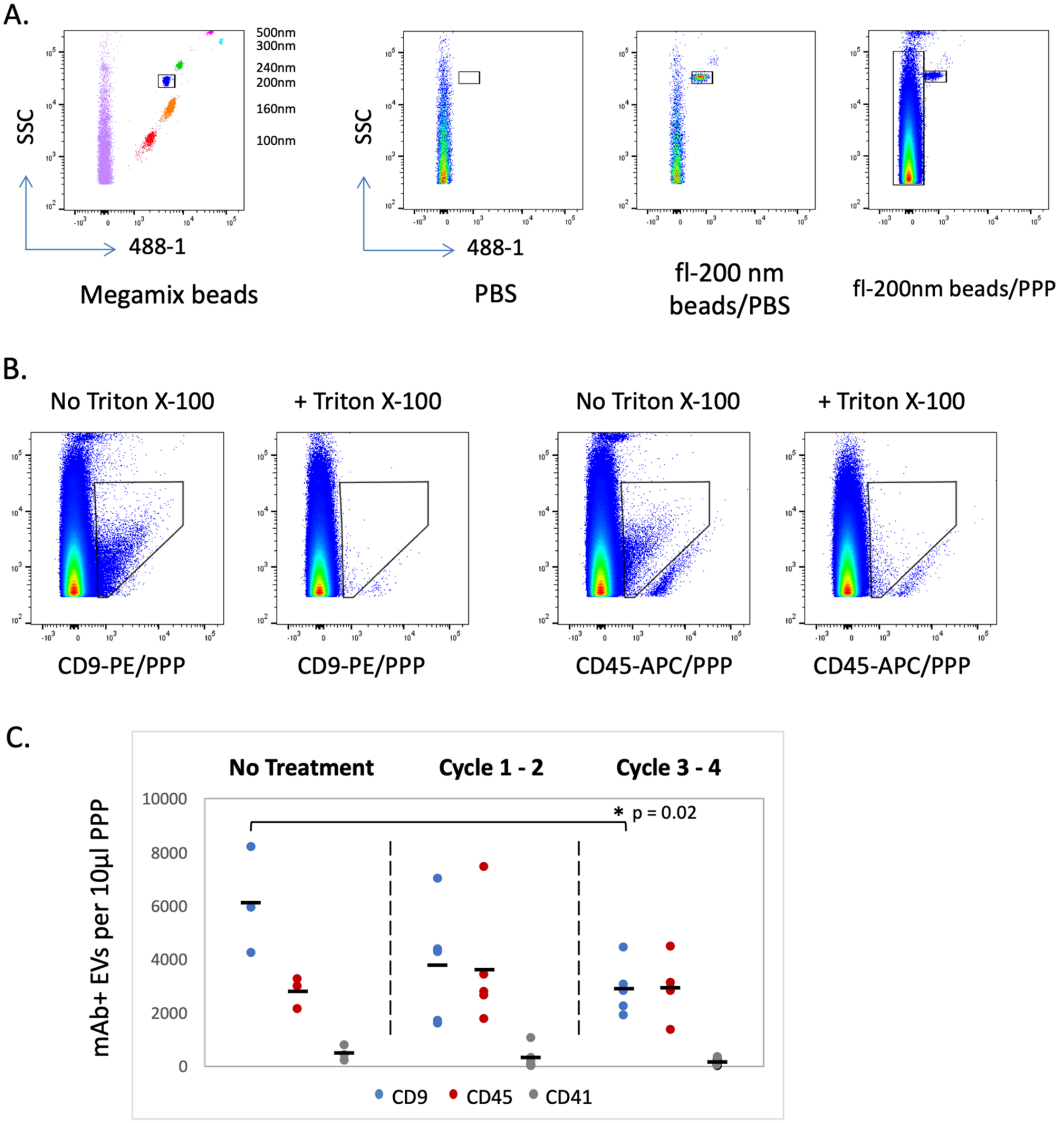
Flow cytometry detection of EVs in PDAC patient plasma. (**A**) Megamix beads (left panel, 100–500 nm in size) were detected with 488 nm fluorescence and were used to standardize the flow cytometer for acquisition of labeled EVs. Additional panels compare acquisition of PBS alone or PBS with 200 nm Fluoresbrite beads (fl-200 nm) or platelet-poor plasma (PPP) with fl-200 nm beads. (**B**) Detection of CD9 and CD45 antibody staining of plasma EVs is reduced to background by the addition of 0.1% Triton X-100. (**C**) CD9, CD45 or CD41 positive EVs were assessed in PPP samples from PDAC patients receiving neoadjuvant treatment for 0 to 4 cycles.

The ability to detect EVs in platelet-poor plasma (PPP) samples from pancreatic cancer patients was investigated. Two types of reference beads were used to ensure instrument consistency and reliability in the detection of EV surface markers by flow cytometry. Megamix fluorescent polystyrene beads, detectable in the 488-1 channel, were used to establish a reference size range on the FACSCanto II cytometer (Fig. 1A). Because the refractive and reflective properties of beads are different from that of EVs^49,50^, reference beads were not used to determine absolute size of the EV population, but rather as a relative reference to establish consistency in the detected size range over the course of this study. For all experiments described here, the same FACSCanto II settings were used. A second reference bead, Fluoresbrite 200 nm beads (fl-200 nm) also detected in the 488-1 channel, was added to each PPP sample prior to analysis to normalize for dilution. Figure 1A shows an example of fl-200 nm beads added to PBS in comparison to a PPP sample. Note the increased nanoparticle density (long box adjacent to fl-200 nm bead box) along the side scatter (SSC) axis in the PPP sample.

For monoclonal antibody (mAb) staining of plasma EV markers, two routinely used EV markers were chosen: CD9 is one of several tetraspanin molecules commonly associated with EVs and CD45 is present on EVs generated by multiple hematopoietic cell types. Figure 1B shows the density plots corresponding to the gating of CD9^+^ or CD45^+^ EVs in a human PPP sample at optimized mAb staining conditions. Also shown in Fig. 1B, treatment with 0.1% Triton X-100 reduced detection of plasma EVs to background levels, verifying that mAb staining was indeed detecting surface markers on vesicles. The detection of CD9^+^ EVs in plasma was further validated by performing size exclusion chromatography (SEC) on plasma samples. SEC fractions were collected after passage over a qEV mini column, and the fractions analyzed by flow cytometry for CD9^+^ EVs. As a general measure of protein contamination, the absorbance at 280 nm was also measured in the same fraction. As shown in Supplemental Fig. S2, CD9^+^ EVs were detected in the expected SEC fractions (fractions #1–3) while proteins detectable by A280 increasingly appeared in later fractions. As further controls on flow cytometry staining of EVs, we demonstrated successful multiparameter staining by co-staining with CD45-specific antibodies in two different channels (Supplemental Fig. S3).

Plasma samples from PDAC patients were surveyed for the detection of EVs using the conditions established above. PDAC samples were grouped according to neoadjuvant chemotherapy treatment duration comparing plasma from patients without treatment to that of patients with increasing cycles of treatment. As seen in Fig. 1C and Supplementary Table S1, CD9 and CD45 EVs were detected in all plasma samples and levels were variable, but CD9 EV levels decreased significantly with more treatment. Average CD9 EV levels detected in PPP samples from untreated patients (6129 ± 1996 EVs/10 µl) were significantly higher than CD9 EV levels in PPP samples after 3–4 cycles of neoadjuvant therapy (2908 ± 978, p = 0.02). While we demonstrate here that EVs were readily detectable in plasma from PDAC patients, interpretation of these results was more difficult. Since many cell types are known to express CD9, it was not possible to establish the source of these plasma EVs or their relationship to disease. Discovery of disease relevant EV markers coupled with tissue-specific EV markers would be of tremendous benefit for diagnosis and treatment evaluation. The platelet marker CD41a was included as a negative control as well as to indicate that samples were handled and processed properly (platelet-poor plasma = low CD41 expression).

### Analysis of EVs produced by PDAC and healthy control pancreatic organoids

Biological fluids, particularly plasma samples, are inherently complex and present a significant challenge for the isolation and characterization of EVs. In addition, many cell types produce EVs, complicating the interpretation of changes in marker levels and source identification. Recent success in the generation and maintenance of organoid cultures provided an opportunity to determine if EVs could be detected in the culture supernatants of pancreatic organoids and enable discovery of more defined PDAC-associated markers.

PDAC and normal pancreatic organoid supernatants were first evaluated for the detection of EVs bearing tetraspanin markers by flow cytometry. Supplemental Fig. S4 shows that EVs positive for tetraspanin markers were detected in pancreatic organoid supernatants and that treatment with Triton X-100 reduced tetraspanin-positive EV detection to background levels. PDAC and healthy control (HC) organoid EVs were screened for CD9, CD63, CD81 and Tspan8 tetraspanin expression and found to be highly variable except for CD81 which showed little to no detection (Supplemental Fig. S5). Heterogeneous presence of tetraspanins was also recently observed in an analysis of exosomes from 14 cell lines of different origins^51^. Interestingly, Tspan8, which we readily detected in both PDAC and HC organoid supernatant EVs, was reported to promote selective uptake into endothelial and pancreatic cells when complexed with integrin α4^52^. CD41a was used as a negative control and was not detected in the supernatant of pancreatic organoids.

**Figure 2:**
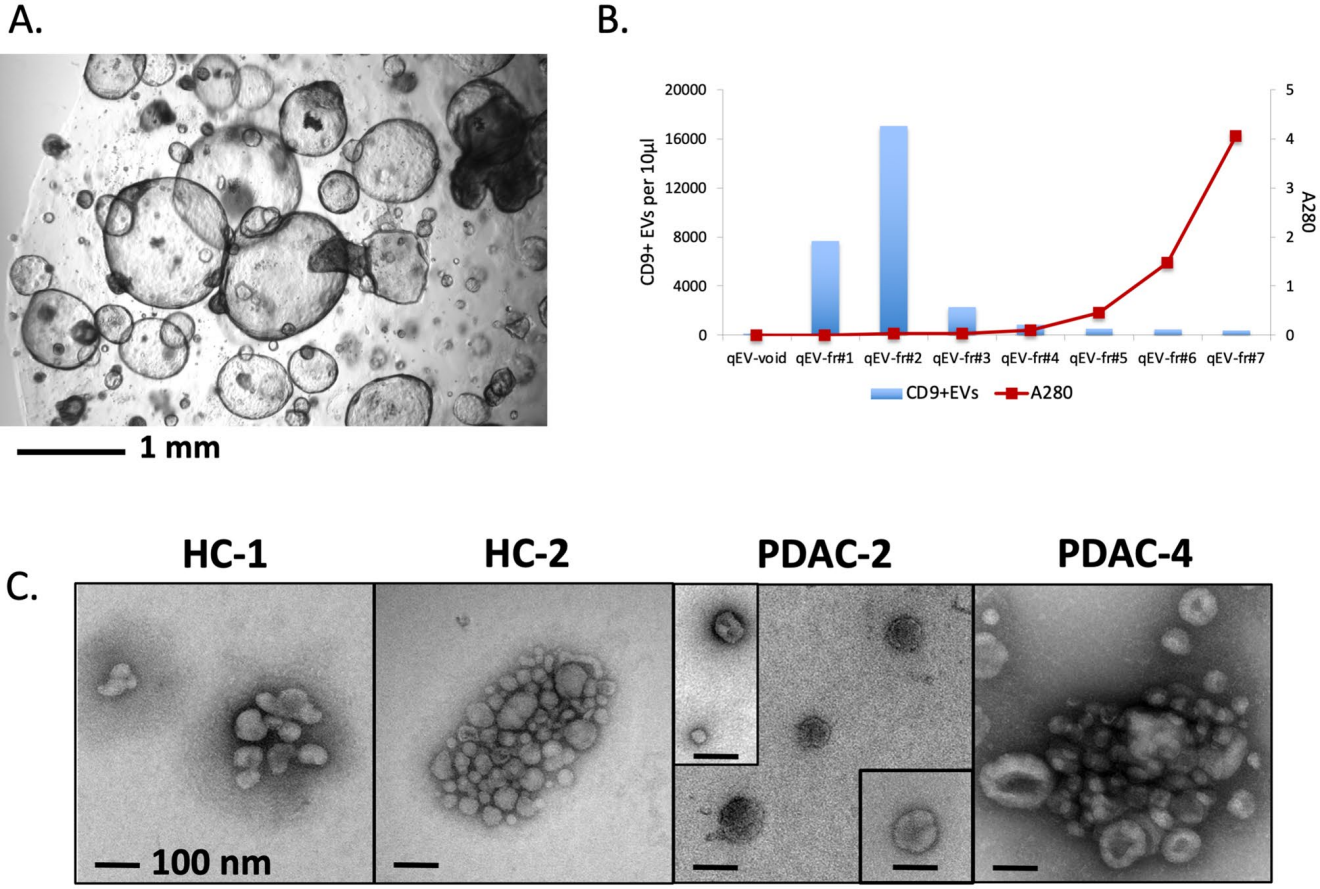
Isolation of EVs from organoids established from PDAC patients and healthy controls (HC). (**A**) Image of representative pancreatic organoid culture (PDAC). (**B**) Fractions obtained after size exclusion chromatography (SEC) of PDAC organoid supernatants were analyzed for CD9^+^ EVs by flow cytometry and protein levels by measurement at 280 nm. (**C**) Representative EM images of EVs isolated after SEC on supernatants collected from two healthy control (HC-1 and HC-2) and two PDAC (PDAC-2 and PDAC-4) organoid cultures.

The use of defined culture media to establish pancreatic organoids (pictured in Fig. 2A) without the presence of serum provided an opportunity to isolate organoid EVs with sufficient purity to provide consistent results by mass spectrometry. Early isolation attempts found that, even with the use of defined media components, bovine serum albumin included in the N2 media supplement presented as a major contaminant in the analysis. Subsequently, a final combination of isolation techniques involving low speed clarification, ultrafiltration and size exclusion chromatography (Fig. 2B) was found to be essential for consistent proteomic analysis without detection of major contaminants. With the adoption of this isolation strategy, proteomic analysis showed that our sample results consistently included many of the top 100 EV proteins reported in the Vesiclepedia database (http://microvesicles.org) and indicates a good enrichment of EV proteins. EM images in Fig. 2C show a sampling of EVs isolated from HC and PDAC pancreatic organoids. Small aggregates of EVs were often observed, which is common at low levels of serum or soluble protein.

### Mass spectrometry of EVs produced by PDAC and healthy control pancreatic organoids

**Figure 3:**
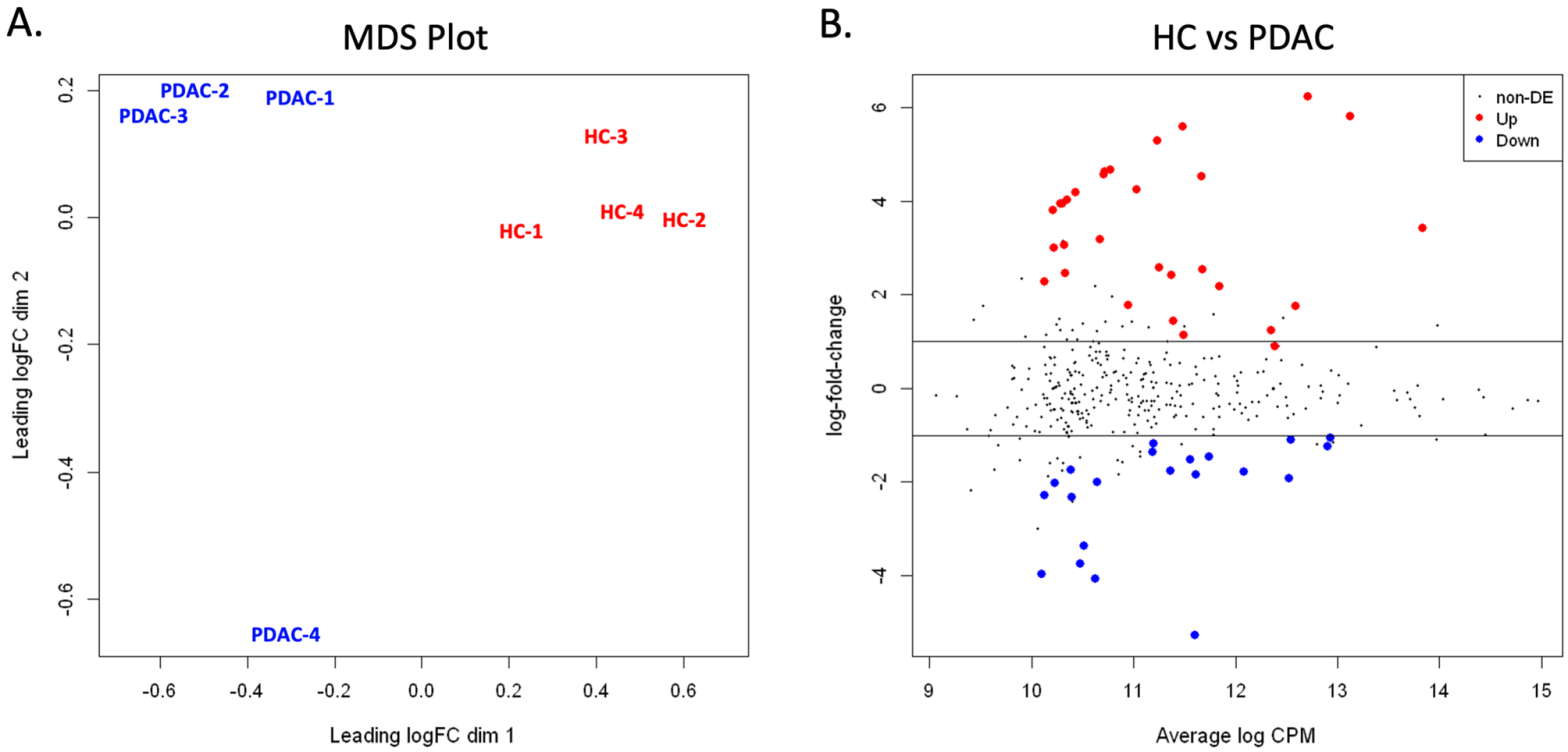
Statistical comparison of proteins identified in EVs isolated from healthy control (HC) and PDAC pancreatic organoids. (**A**) Multidimensional scaling (MDS) demonstrates clustering of samples by condition (HC versus PDAC) with PDAC-4 further removed from other PDAC samples. (**B**) Distribution of the candidate proteins with significant change (Up or Down) or without differential expression (non-DE) between the HC and PDAC sample sets are visualized by plotting fold change vs CPM.

An experiment was set up for comparative proteomic analysis of supernatant EVs isolated from 4 individual PDAC organoid cultures versus 4 HC pancreatic organoid cultures (4 × 4 study). Supernatant EVs were isolated and processed in an identical manner, then equivalent amounts of sample were analyzed in parallel by mass spectrometry (see “Methods”). Protein candidates for differential expression were analyzed for significant change between the HC and PDAC sample sets. A multidimensional scaling (MDS) plot of the normalized data (Fig. 3A) showed clustering of the samples by condition, indicating good separation of HC and PDAC samples. Figure 3B shows the data distribution of HC over PDAC candidates with fold-change plotted against CPM, where UP regulated proteins are more highly expressed in HC EVs and DOWN regulated proteins are more highly expressed in PDAC EVs. Fifty-one candidates were identified as being up or down regulated at a < 5% false discovery rate (FDR, p-value < 0.05) while approximately 320 proteins showed no significant change (non-DE).

**Figure 4:**
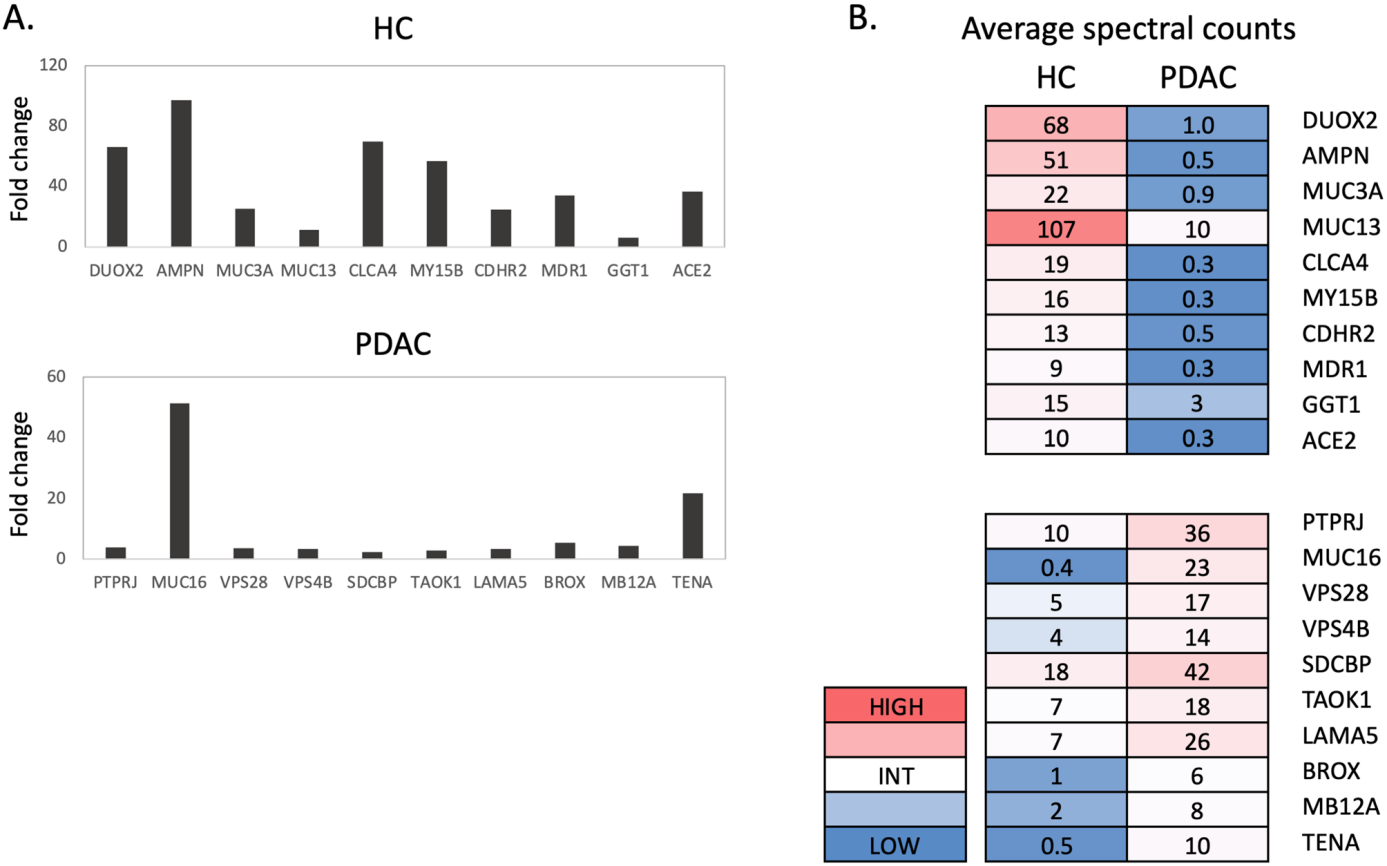
Proteomic analysis of EV samples isolated from four healthy control (HC) and four pancreatic cancer (PDAC) organoid cultures (4 × 4 study). (**A**) Fold change of spectral count averages for top 10 proteins upregulated in HC (top panel) and top 10 proteins upregulated in PDAC (bottom panel). (**B**) Spectral count averages in the HC or PDAC group for each corresponding protein identified in (**A**).

Figure 4A lists the top ten proteins identified with the greatest positive fold change, representing proteins differentially upregulated in HC EVs (top panel). Conversely, also listed are the ten proteins with the greatest negative fold change, representing proteins differentially upregulated in PDAC EVs (bottom panel). The results for these individual samples instead of group averages are provided in Supplementary Table S7. Interestingly, protein fold changes observed for the top ten proteins in HC EVs were considerably higher compared to the top ten proteins in PDAC EVs. Proteins in the HC group were at levels 10- to 90-fold greater than in the PDAC group, while this magnitude of difference was not observed with upregulated proteins in the PDAC group.

Average spectral count for each of these proteins is shown in Fig. 4B. In choosing a screening marker for the detection of EVs in plasma for example, the presence of more abundant EV proteins would much improve the signal to noise ratio. Thus, a more detectable signal as well as greater fold difference between PDAC versus healthy samples is desirable in choosing candidate screening markers. Based on these criteria, the 2 best marker candidates in the top 10 list of the HC group are DUOX2 and AMPN. MUC3A and CLCA4 are also reasonable candidates although abundance levels in the HC samples were more variable (Supplementary Table S7). The choices for top candidate markers in the PDAC group were made more difficult by the variable levels seen in individual PDAC EV samples (Supplementary Table S7). Potential markers chosen from this list include PTPRJ, MUC16, SDCBP and LAMA5.

### Protein interaction analysis

**Figure 5:**
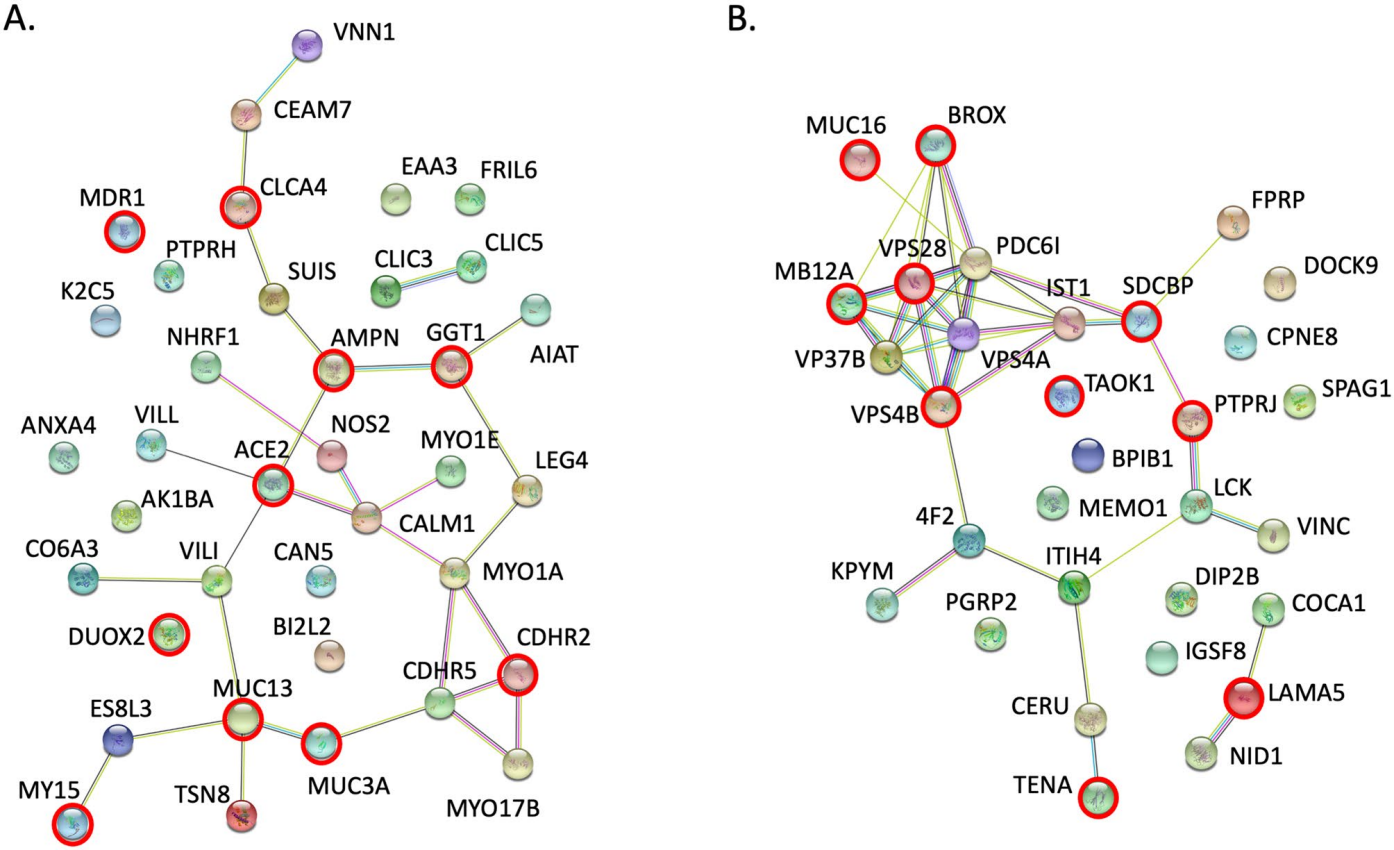
Protein interaction analysis. The STRING database (https://string-db.org) was used to analyze protein–protein interactions among the candidates found to be upregulated in pancreatic organoid EVs from (**A**) healthy controls or (**B**) PDAC patients. Nodes highlighted with a red ring designate proteins within the top ten highest fold change in each group (p < 0.01, see Fig. 4).

Protein–protein interactions between the upregulated proteins identified in HC or PDAC organoid EVs were investigated using the STRING database for functional protein association networks (https://string-db.org, version 11.0). For this purpose, additional candidate proteins with < 10% FDR were included. Figure 5A included the network mapping of 37 proteins upregulated in HC organoid EVs with the top 10 proteins (< 1% FDR) highlighted in a red circle. Gene ontology (GO) analysis matched 16 of 37 proteins to functions ‘regulating biological quality’ (see Supplementary Table S8). Other top processes included ‘ion transport’ (9/37) and ‘regulation of response to stress’ (9/37). Thus, more abundant proteins found in HC EVs were involved in cell maintenance and response to environment.

Figure 5B shows the mapping of 31 protein candidates upregulated in PDAC organoid EVs with the top 10 proteins circled in red. A cluster of proteins involved in the sorting and transport of vesicles through the exosome/MVB pathway was evident, with identification of 3 of the 4 proteins that comprise the ESCRT-I tetramer complex^53^. Top 10 candidates also included proteins associated with tumor progression (LAMA5, SDCBP, TENA, PTPRJ, MUC16, TAOK1; see “Discussion”). Top matches in the GO analysis of PDAC EVs included ‘cellular component organization’ (18/31), ‘localization’ (18/31), and ‘transport’ (16/31) (Supplementary Table S9). Processes specifically relevant to EVs included ‘vesicle-mediated transport’ (12/31) and ‘multivesicular body assembly’ (8/31). Thus, several abundant proteins found in PDAC EVs involved transport molecules, particularly those associated with the exosome/MVB transport process.

### Extension of EV proteomic results to additional PDAC EV samples

**Figure 6:**
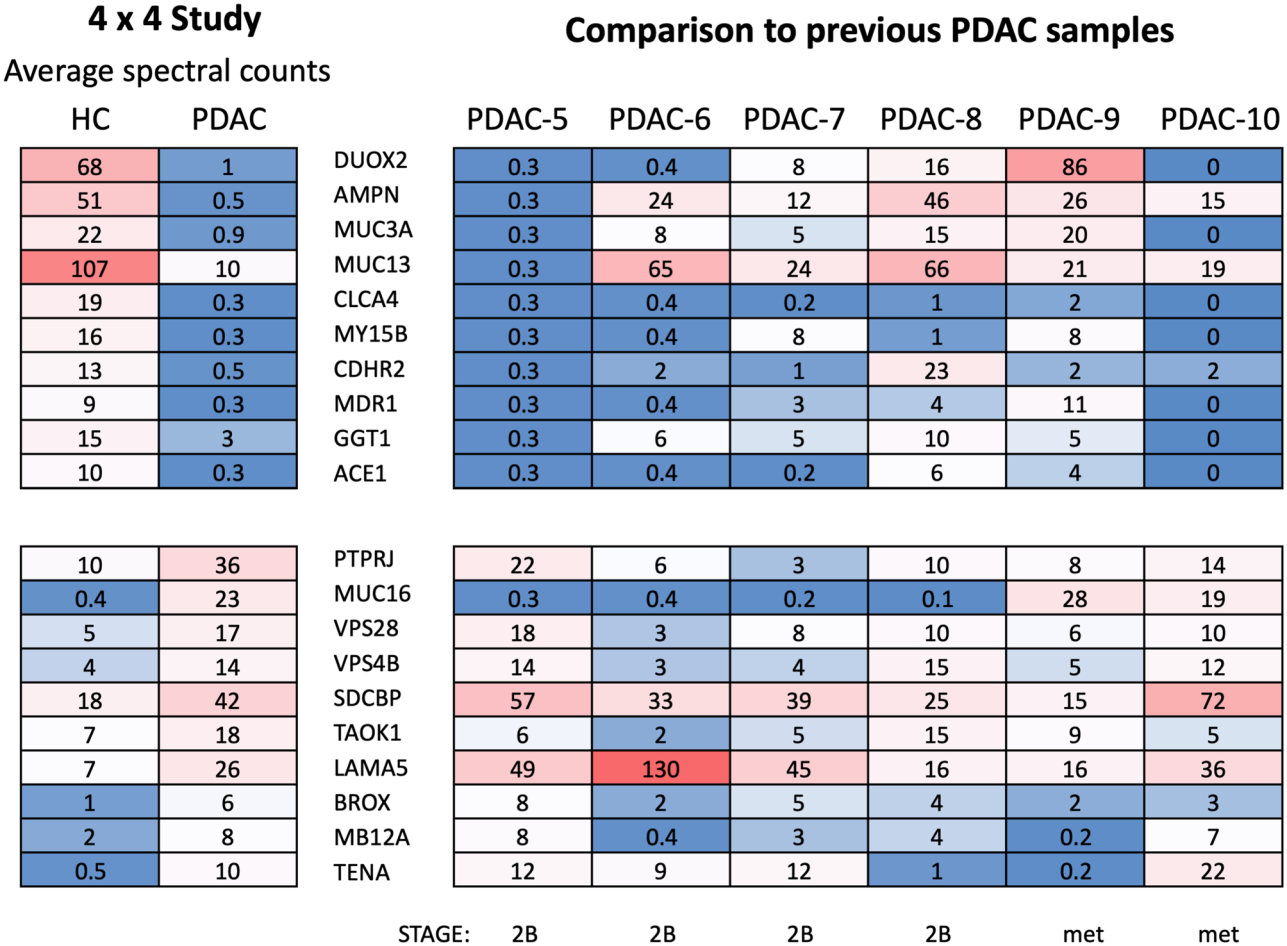
Upregulated proteins analyzed in additional PDAC organoid EVs. Comparison of average spectral counts of top proteins identified in the 4 × 4 study (see Fig. 4) to normalized spectral counts for each corresponding protein in six additional PDAC organoid EV samples. PDAC stage is shown for each PDAC organoid (*met* metastatic tumor sample).

Results from the top candidates generated by the 4 × 4 study described above were retrospectively compared to proteomic results of EV samples generated from a larger panel of six PDAC organoid cultures that were processed in an identical manner. Supplemental Fig. S6 shows the fold change vs CPM distribution of up and down regulated proteins for these groups. As can be seen from spectral counts compared in Fig. 6, the detection of most candidate markers in this panel of six PDAC samples was more variable than results seen with the 4 × 4 study. Among the protein candidates upregulated in the 4 × 4 HC EVs, only CLCA4 was minimally present in all six additional PDAC EV samples. Among the 4 × 4 PDAC upregulated proteins, only LAMA5 consistently showed increased levels in all six additional PDAC samples. SDCBP and TENA were upregulated in 5/6 and 4/6 of the additional PDAC samples, respectively. This is a reasonable finding given the large potential for variability among PDAC samples. Some common variables would include disease stage, tumor location and microenvironment, immune responsiveness and/or drug treatment prior to resection. Differences in proteomic results did not correlate with disease stage at the time of tumor collection in this study. However, the generation of a candidate marker list is a significant step forward in the identification of EV markers capable of differentiating between PDAC and healthy individuals. Several candidate proteins identified in this study from PDAC EVs have been reported in multiple types of cancer (see “Discussion”).

## Discussion

A rapidly increasing number of EV studies are being published and are greatly expanding the diversity of roles assigned to these biological complexes. The production of EVs by most cells, once thought to be a mechanism for eliminating waste material, is now considered to be an important means for intercellular communication as well as local and long-distance delivery of bioactive molecules. EVs produced by malignant cells are very likely to bear unique profiles resulting from genetic mutation or deregulated protein levels. Such EVs also act to promote tumorigenesis by altering the microenvironment and suppressing local immune function. An in-depth analysis of cancer associated EVs therefore provides an opportunity both to understand the biology of cancer progression and to identify markers of disease. In the case of PDAC, a discovery of early disease changes in selected EV markers could substantially decrease the rate of mortality.

The detection and analysis of isolated EVs is challenging on multiple levels. EVs released into tissue or the microenvironment may be short-lived while EVs present in complex biofluids are difficult to isolate in sufficient amounts without non-specific contaminants. In this current study we detected EVs in PDAC patient plasma and in supernatants from PDAC and HC pancreatic organoid cultures using high resolution flow cytometry. Flow cytometry detection of tetraspanins was advantageous for detection purposes but cannot be used to determine cell type or tissue source of EVs in most biological fluids. Identification of cell-type-specific EV markers for use in multiparameter screening of EVs would greatly improve detection as a diagnostic tool.

The isolation of EVs produced in cell culture is an alternative strategy that circumvents many of the complexities associated with the analysis of EVs in plasma samples. EVs from pancreatic cell lines grown in 2D culture have been analyzed for protein biomarkers^54,55^, with little overlap to our study except for the upregulation of LAMA5^55^. 3D organoid cultures provide the added advantage of recapitulating complex cellular interactions and self-renewal capacity. A recent study investigated the miRNA cargo of EVs derived from PDAC organoids^56^; potential biomarkers were also reported in a study of organoids derived from PDAC xenografts^57^ but were not found to overlap with markers identified in our study. After adjustment of isolation procedures in our study, EVs suitable for mass spectrometry were isolated from PDAC and healthy pancreatic organoid cultures. In total, we carried out proteomic analysis on EVs isolated from 10 PDAC organoid cultures and 4 healthy control pancreatic organoid cultures. These samples were from a defined tissue source with minimal contamination from human serum proteins or lipoproteins. PDAC organoids were established from resected tumor tissue at various stages of disease, which may have contributed to variable marker levels seen in the results. In our 4 × 4 study, we identified proteins upregulated in either HC EVs or PDAC EVs. Proteins upregulated in HC EVs play significant roles in maintaining cellular homeostasis. Additionally, some are reportedly tumor suppressive: (DUOX2^58^, MUC3A^59,60^, CLCA4^61–63^, CDHR2^64,65^. In contrast, proteins upregulated in PDAC EVs were associated with tumor progression or were part of the exosome/MVB sorting and release machinery. The latter indicates that increased output of EVs specifically associated with the exosome/MVB pathway occurs in PDAC tumor cells and that some components may remain within EVs. Alternatively, it is possible that these proteins play some role in uptake or delivery at the target site, although no such role for ESCRT-I proteins or VSP4A/B has been reported. It is interesting that only proteins in the ESCRT-I complex (MB12A, VPS28, VP37B) and the ATPase release protein (VPS4A, VPS4B), but not the ESCRT-II or -III components, were identified. It is possible that select components of this pathway remain associated with exosomes as they are overexpressed in PDAC cells.

Of the top upregulated proteins identified in PDAC EVs, three proteins are notable for their reported role in cancer progression. (1) Laminin subunit alpha 5 (LAMA5), an extracellular matrix glycoprotein, was consistently elevated in all PDAC organoid EV samples analyzed here. In colorectal cancer, LAMA5 promoted branching angiogenesis and liver metastasis. Additional studies found that LAMA5 played a role in the interaction of colorectal tumor cells with endothelium and that LAMA5 knockdown in colorectal cancer cells affected differentiation and sensitivity to drug treatment^66–69^. Interestingly, using mass spectrometry we also identified LAMA5 to be a highly elevated protein in EVs isolated from a mouse pancreatic tumor line (ACB, unpublished) that is used extensively as an orthotopic murine model of PDAC^70^. (2) Syntenin1 (SDCBP), also known as melanoma differentiation-associated protein-9 (MDA-9), is an adapter molecule that regulates multiple membrane-associated processes, including exosome biogenesis, by binding various partner molecules. SDCBP also regulates cell membrane motility and promoted tumor growth and metastasis in melanoma, prostate, breast and gastric cancer cells^71,72^. In a recent paper, Kugeratski et al.^51^ identified syntenin-1 as the most consistently abundant protein in exosomes isolated from 14 cell lines, and the use of syntenin-1 as a universal marker for exosomes was proposed. With regard to our own current study, the upregulated expression of syntenin-1 in PDAC EVs is consistent with a shift to increased exosome biogenesis during cancer progression. (3) Tenascin (TENA), also known as glioma-associated extracellular matrix antigen, is an extracellular matrix glycoprotein that regulates cell adhesion and movement in development and injury repair. TENA promotes angiogenesis and is associated with poor prognosis in several cancer types; downregulation of TENA also inhibited breast cancer growth and migration^73–75^. Additionally, TENA plays a role in neurite outgrowth and guidance, and was found to promote concomitant outgrowth between PDAC and neuronal cells in co-culture^76^. This is of particular interest in PDAC, where perineural invasion is common. Finally, other proteins identified in our 4 × 4 proteomic comparison of PDAC vs HC organoid EVs with more variability in the larger PDAC panel (PTPRJ, MUC16, TAOK1) are also reported markers of tumor-development in several cancer models^77–79^.

The variable level of these protein markers also likely reflects the source heterogeneity in our PDAC organoid samples. Intrinsic factors of the pancreatic cancer and tumor microenvironment may contribute to this variability, as well as disease stage and treatment. Subset analysis of numerous additional samples will be required to address the issue of variability. In addition, candidate proteins for PDAC screening will need to be validated by independent study.

In conclusion, the results from this study are highly encouraging in terms of the potential markers identified from the EV populations of both PDAC and HC organoids. The EV protein profiles from PDAC versus HC organoids were distinct and associated with different cellular programs consistent with a tumorigenic or healthy state. Through careful selection of screening markers using pancreatic organoids, one may design a panel to detect pancreatic EVs in plasma that display a malignant or healthy profile to indicate the presence of ongoing disease.

## Supplementary Information

Supplementary Figure S1.

Supplementary Figure S2.

Supplementary Figure S3.

Supplementary Figure S4.

Supplementary Figure S5.

Supplementary Figure S6.

Supplementary Table S1.

Supplementary Table S2.

Supplementary Table S3.

Supplementary Table S4.

Supplementary Table S5.

Supplementary Table S6.

Supplementary Table S7.

Supplementary Table S8.

Supplementary Table S9.

## Data Availability

The datasets generated during the current study are available from the corresponding author on reasonable request.

## Abbreviations

EV: Extracellular vesicles
MVB: Multivesicular body
PPP: Platelet-poor plasma
PDAC: Pancreatic ductal adenocarcinoma
HC: Healthy control
HRFC: High resolution flow cytometry
SEC: Size exclusion chromatography
PE: Phycoerythrin
APC: Allophycocyanin
AF647: Alexa Fluor 647

## References

[1] Berumen Sanchez G, Bunn KE, Pua HH, Rafat M (2021). Extracellular vesicles: Mediators of intercellular communication in tissue injury and disease. Cell Commun. Signal..

[2] Pitt JM, Kroemer G, Zitvogel L (2016). Extracellular vesicles: Masters of intercellular communication and potential clinical interventions. J. Clin. Investig..

[3] Hoshino A, Kim HS, Bojmar L, Gyan KE, Cioffi M, Hernandez J (2020). Extracellular vesicle and particle biomarkers define multiple human cancers. Cell.

[4] Becker A, Thakur BK, Weiss JM, Kim HS, Peinado H, Lyden D (2016). Extracellular vesicles in cancer: Cell-to-cell mediators of metastasis. Cancer Cell.

[5] Pink RC, Elmusrati AA, Lambert D, Carter DRF (2018). Royal Society Scientific Meeting: Extracellular vesicles in the tumour microenvironment. Philos. Trans. R. Soc. Lond. B Biol. Sci..

[6] Zhao H, Achreja A, Iessi E, Logozzi M, Mizzoni D, Di Raimo R (2018). The key role of extracellular vesicles in the metastatic process. Biochim. Biophys. Acta Rev. Cancer.

[7] Czernek L, Duchler M (2017). Functions of cancer-derived extracellular vesicles in immunosuppression. Arch. Immunol. Ther. Exp. (Warsz).

[8] Dorsam B, Reiners KS, von Strandmann EP (2018). Cancer-derived extracellular vesicles: Friend and foe of tumour immunosurveillance. Philos. Trans. R. Soc. Lond. B Biol. Sci..

[9] Guo C, Liu J, Zhou Q, Song J, Zhang Z, Li Z (2020). Exosomal noncoding RNAs and tumor drug resistance. Cancer Res..

[10] Han L, Xu J, Xu Q, Zhang B, Lam EW, Sun Y (2017). Extracellular vesicles in the tumor microenvironment: Therapeutic resistance, clinical biomarkers, and targeting strategies. Med. Res. Rev..

[11] Samuel P, Fabbri M, Carter DRF (2017). Mechanisms of drug resistance in cancer: The role of extracellular vesicles. Proteomics.

[12] Santos P, Almeida F (2020). Role of exosomal miRNAs and the tumor microenvironment in drug resistance. Cells.

[13] Armstrong EA, Beal EW, Chakedis J, Paredes AZ, Moris D, Pawlik TM (2018). Exosomes in pancreatic cancer: From early detection to treatment. J. Gastrointest. Surg..

[14] Yan Y, Fu G, Ming L (2018). Role of exosomes in pancreatic cancer. Oncol. Lett..

[15] Yu Z, Zhao S, Ren L, Wang L, Chen Z, Hoffman RM (2017). Pancreatic cancer-derived exosomes promote tumor metastasis and liver pre-metastatic niche formation. Oncotarget.

[16] Ali S, Suresh R, Banerjee S, Bao B, Xu Z, Wilson J (2015). Contribution of microRNAs in understanding the pancreatic tumor microenvironment involving cancer associated stellate and fibroblast cells. Am. J. Cancer Res..

[17] Leca J, Martinez S, Lac S, Nigri J, Secq V, Rubis M (2016). Cancer-associated fibroblast-derived annexin A6+ extracellular vesicles support pancreatic cancer aggressiveness. J. Clin. Investig..

[18] Richards KE, Zeleniak AE, Fishel ML, Wu J, Littlepage LE, Hill R (2017). Cancer-associated fibroblast exosomes regulate survival and proliferation of pancreatic cancer cells. Oncogene.

[19] Takikawa T, Masamune A, Yoshida N, Hamada S, Kogure T, Shimosegawa T (2017). Exosomes derived from pancreatic stellate cells: MicroRNA signature and effects on pancreatic cancer cells. Pancreas.

[20] Whiteside TL (2017). Profiling of plasma-derived extracellular vesicles cargo for diagnosis of pancreatic malignancy. Ann. Transl. Med..

[21] Frampton AE, Prado MM, Lopez-Jimenez E, Fajardo-Puerta AB, Jawad ZAR, Lawton P (2018). Glypican-1 is enriched in circulating-exosomes in pancreatic cancer and correlates with tumor burden. Oncotarget.

[22] Melo SA, Luecke LB, Kahlert C, Fernandez AF, Gammon ST, Kaye J (2015). Glypican-1 identifies cancer exosomes and detects early pancreatic cancer. Nature.

[23] Allenson K, Castillo J, San Lucas FA, Scelo G, Kim DU, Bernard V (2017). High prevalence of mutant KRAS in circulating exosome-derived DNA from early-stage pancreatic cancer patients. Ann. Oncol..

[24] Yang S, Che SP, Kurywchak P, Tavormina JL, Gansmo LB, Correa de Sampaio P (2017). Detection of mutant KRAS and TP53 DNA in circulating exosomes from healthy individuals and patients with pancreatic cancer. Cancer Biol. Ther..

[25] Lai X, Wang M, McElyea SD, Sherman S, House M, Korc M (2017). A microRNA signature in circulating exosomes is superior to exosomal glypican-1 levels for diagnosing pancreatic cancer. Cancer Lett..

[26] Patel GK, Khan MA, Bhardwaj A, Srivastava SK, Zubair H, Patton MC (2017). Exosomes confer chemoresistance to pancreatic cancer cells by promoting ROS detoxification and miR-155-mediated suppression of key gemcitabine-metabolising enzyme, DCK. Br. J. Cancer.

[27] Yang Z, Zhao N, Cui J, Wu H, Xiong J, Peng T (2020). Exosomes derived from cancer stem cells of gemcitabine-resistant pancreatic cancer cells enhance drug resistance by delivering miR-210. Cell Oncol. (Dordr)..

[28] Madhavan B, Yue S, Galli U, Rana S, Gross W, Muller M (2015). Combined evaluation of a panel of protein and miRNA serum-exosome biomarkers for pancreatic cancer diagnosis increases sensitivity and specificity. Int. J. Cancer.

[29] Ramirez MI, Amorim MG, Gadelha C, Milic I, Welsh JA, Freitas VM (2018). Technical challenges of working with extracellular vesicles. Nanoscale.

[30] Boing AN, van der Pol E, Grootemaat AE, Coumans FA, Sturk A, Nieuwland R (2014). Single-step isolation of extracellular vesicles by size-exclusion chromatography. J. Extracell. Vesicles.

[31] Brennan K, Martin K, FitzGerald SP, O'Sullivan J, Wu Y, Blanco A (2020). A comparison of methods for the isolation and separation of extracellular vesicles from protein and lipid particles in human serum. Sci. Rep..

[32] Yuana Y, Levels J, Grootemaat A, Sturk A, Nieuwland R (2014). Co-isolation of extracellular vesicles and high-density lipoproteins using density gradient ultracentrifugation. J. Extracell. Vesicles.

[33] Nolte-'t Hoen EN, van der Vlist EJ, Aalberts M, Mertens HC, Bosch BJ, Bartelink W (2012). Quantitative and qualitative flow cytometric analysis of nanosized cell-derived membrane vesicles. Nanomedicine.

[34] Lotvall J, Hill AF, Hochberg F, Buzas EI, Di Vizio D, Gardiner C (2014). Minimal experimental requirements for definition of extracellular vesicles and their functions: A position statement from the International Society for Extracellular Vesicles. J. Extracell. Vesicles.

[35] Thery C, Witwer KW, Aikawa E, Alcaraz MJ, Anderson JD, Andriantsitohaina R (2018). Minimal information for studies of extracellular vesicles 2018 (MISEV2018): A position statement of the International Society for Extracellular Vesicles and update of the MISEV2014 guidelines. J. Extracell. Vesicles.

[36] Witwer KW, Soekmadji C, Hill AF, Wauben MH, Buzas EI, Di Vizio D (2017). Updating the MISEV minimal requirements for extracellular vesicle studies: Building bridges to reproducibility. J. Extracell. Vesicles.

[37] Moreira L, Bakir B, Chatterji P, Dantes Z, Reichert M, Rustgi AK (2018). Pancreas 3D organoids: Current and future aspects as a research platform for personalized medicine in pancreatic cancer. Cell. Mol. Gastroenterol. Hepatol..

[38] Dorrell C, Schug J, Lin CF, Canaday PS, Fox AJ, Smirnova O (2011). Transcriptomes of the major human pancreatic cell types. Diabetologia.

[39] Dorrell C, Tarlow B, Wang Y, Canaday PS, Haft A, Schug J (2014). The organoid-initiating cells in mouse pancreas and liver are phenotypically and functionally similar. Stem Cell Res..

[40] Welsh JA, Van Der Pol E, Arkesteijn GJA, Bremer M, Brisson A, Coumans F (2020). MIFlowCyt-EV: A framework for standardized reporting of extracellular vesicle flow cytometry experiments. J. Extracell. Vesicles.

[41] Nolan JP, Duggan E (2018). Analysis of individual extracellular vesicles by flow cytometry. Methods Mol. Biol..

[42] van der Pol E, van Gemert MJ, Sturk A, Nieuwland R, van Leeuwen TG (2012). Single vs. swarm detection of microparticles and exosomes by flow cytometry. J. Thromb. Haemost..

[43] Osteikoetxea X, Sodar B, Nemeth A, Szabo-Taylor K, Paloczi K, Vukman KV (2015). Differential detergent sensitivity of extracellular vesicle subpopulations. Org. Biomol. Chem..

[44] Eng JK, Jahan TA, Hoopmann MR (2013). Comet: An open-source MS/MS sequence database search tool. Proteomics.

[45] Wilmarth PA, Riviere MA, David LL (2009). Techniques for accurate protein identification in shotgun proteomic studies of human, mouse, bovine, and chicken lenses. J. Ocul. Biol. Dis. Inform..

[46] Keller A, Nesvizhskii AI, Kolker E, Aebersold R (2002). Empirical statistical model to estimate the accuracy of peptide identifications made by MS/MS and database search. Anal. Chem..

[47] Robinson MD, McCarthy DJ, Smyth GK (2010). edgeR: A Bioconductor package for differential expression analysis of digital gene expression data. Bioinformatics.

[48] Perez-Riverol Y, Csordas A, Bai J, Bernal-Llinares M, Hewapathirana S, Kundu DJ (2019). The PRIDE database and related tools and resources in 2019: Improving support for quantification data. Nucleic Acids Res..

[49] Chandler WL, Yeung W, Tait JF (2011). A new microparticle size calibration standard for use in measuring smaller microparticles using a new flow cytometer. J. Thromb. Haemost..

[50] Parida BK, Garrastazu H, Aden JK, Cap AP, McFaul SJ (2015). Silica microspheres are superior to polystyrene for microvesicle analysis by flow cytometry. Thromb. Res..

[51] Kugeratski FG, Hodge K, Lilla S, McAndrews KM, Zhou X, Hwang RF (2021). Quantitative proteomics identifies the core proteome of exosomes with syntenin-1 as the highest abundant protein and a putative universal biomarker. Nat. Cell. Biol..

[52] Rana S, Yue S, Stadel D, Zoller M (2012). Toward tailored exosomes: The exosomal tetraspanin web contributes to target cell selection. Int. J. Biochem. Cell Biol..

[53] Teis D, Saksena S, Emr SD (2009). SnapShot: The ESCRT machinery. Cell.

[54] Fahrmann JF, Mao X, Irajizad E, Katayama H, Capello M, Tanaka I (2020). Plasma-derived extracellular vesicles convey protein signatures that reflect pathophysiology in lung and pancreatic adenocarcinomas. Cancers (Basel).

[55] Guerreiro EM, Ovstebo R, Thiede B, Costea DE, Soland TM, Kanli GH (2020). Cancer cell line-specific protein profiles in extracellular vesicles identified by proteomics. PLoS One.

[56] Zeold A, Sandor GO, Kiss A, Soos AA, Tolgyes T, Bursics A (2021). Shared extracellular vesicle miRNA profiles of matched ductal pancreatic adenocarcinoma organoids and blood plasma samples show the power of organoid technology. Cell Mol. Life Sci..

[57] Huang L, Bockorny B, Paul I, Akshinthala D, Frappart PO, Gandarilla O (2020). PDX-derived organoids model in vivo drug response and secrete biomarkers. JCI Insight.

[58] Gibson AR, O'Leary BR, Du J, Sarsour EH, Kalen AL, Wagner BA (2020). Dual oxidase-induced sustained generation of hydrogen peroxide contributes to pharmacologic ascorbate-induced cytotoxicity. Cancer Res..

[59] Buisine MP, Devisme L, Degand P, Dieu MC, Gosselin B, Copin MC (2000). Developmental mucin gene expression in the gastroduodenal tract and accessory digestive glands. II. Duodenum and liver, gallbladder, and pancreas. J. Histochem. Cytochem..

[60] Giuntoli RL, Rodriguez GC, Whitaker RS, Dodge R, Voynow JA (1998). Mucin gene expression in ovarian cancers. Cancer Res..

[61] Liu Z, Chen M, Xie LK, Liu T, Zou ZW, Li Y (2018). CLCA4 inhibits cell proliferation and invasion of hepatocellular carcinoma by suppressing epithelial-mesenchymal transition via PI3K/AKT signaling. Aging (Albany NY).

[62] Chen H, Liu Y, Jiang CJ, Chen YM, Li H, Liu QA (2019). Calcium-activated chloride channel A4 (CLCA4) plays inhibitory roles in invasion and migration through suppressing epithelial-mesenchymal transition via PI3K/AKT signaling in colorectal cancer. Med. Sci. Monit..

[63] Yu Y, Walia V, Elble RC (2013). Loss of CLCA4 promotes epithelial-to-mesenchymal transition in breast cancer cells. PLoS One.

[64] Crawley SW, Shifrin DA, Grega-Larson NE, McConnell RE, Benesh AE, Mao S (2014). Intestinal brush border assembly driven by protocadherin-based intermicrovillar adhesion. Cell.

[65] Okazaki N, Takahashi N, Kojima S, Masuho Y, Koga H (2002). Protocadherin LKC, a new candidate for a tumor suppressor of colon and liver cancers, its association with contact inhibition of cell proliferation. Carcinogenesis.

[66] Bartolini A, Cardaci S, Lamba S, Oddo D, Marchio C, Cassoni P (2016). BCAM and LAMA5 mediate the recognition between tumor cells and the endothelium in the metastatic spreading of KRAS-mutant colorectal cancer. Clin. Cancer Res..

[67] Gordon-Weeks A, Lim SY, Yuzhalin A, Lucotti S, Vermeer JAF, Jones K (2019). Tumour-derived laminin alpha5 (LAMA5) promotes colorectal liver metastasis growth, branching angiogenesis and notch pathway inhibition. Cancers (Basel)..

[68] Kikkawa Y, Ogawa T, Sudo R, Yamada Y, Katagiri F, Hozumi K (2013). The lutheran/basal cell adhesion molecule promotes tumor cell migration by modulating integrin-mediated cell attachment to laminin-511 protein. J. Biol. Chem..

[69] Kusuma N, Denoyer D, Eble JA, Redvers RP, Parker BS, Pelzer R (2012). Integrin-dependent response to laminin-511 regulates breast tumor cell invasion and metastasis. Int. J. Cancer.

[70] Michaelis KA, Zhu X, Burfeind KG, Krasnow SM, Levasseur PR, Morgan TK (2017). Establishment and characterization of a novel murine model of pancreatic cancer cachexia. J. Cachexia Sarcopenia Muscle.

[71] Kashyap R, Roucourt B, Lembo F, Fares J, Carcavilla AM, Restouin A (2015). Syntenin controls migration, growth, proliferation, and cell cycle progression in cancer cells. Front. Pharmacol..

[72] Koo TH, Lee JJ, Kim EM, Kim KW, Kim HD, Lee JH (2002). Syntenin is overexpressed and promotes cell migration in metastatic human breast and gastric cancer cell lines. Oncogene.

[73] Angel I, Pilo Kerman O, Rousso-Noori L, Friedmann-Morvinski D (2020). Tenascin C promotes cancer cell plasticity in mesenchymal glioblastoma. Oncogene.

[74] Wawrzyniak D, Grabowska M, Glodowicz P, Kuczynski K, Kuczynska B, Fedoruk-Wyszomirska A (2020). Down-regulation of tenascin-C inhibits breast cancer cells development by cell growth, migration, and adhesion impairment. PLoS One.

[75] Yalcin F, Dzaye O, Xia S (2020). Tenascin-C function in glioma: Immunomodulation and beyond. Adv. Exp. Med. Biol..

[76] Furuhashi S, Sakaguchi T, Murakami T, Fukushima M, Morita Y, Ikegami K (2020). Tenascin C in the tumor-nerve microenvironment enhances perineural invasion and correlates with locoregional recurrence in pancreatic ductal adenocarcinoma. Pancreas.

[77] Chen X, Li X, Wang X, Zhu Q, Wu X, Wang X (2019). MUC16 impacts tumor proliferation and migration through cytoplasmic translocation of P120-catenin in epithelial ovarian cancer cells: An original research. BMC Cancer.

[78] Koo CY, Giacomini C, Reyes-Corral M, Olmos Y, Tavares IA, Marson CM (2017). Targeting TAO kinases using a new inhibitor compound delays mitosis and induces mitotic cell death in centrosome amplified breast cancer cells. Mol. Cancer Ther..

[79] Spring K, Fournier P, Lapointe L, Chabot C, Roussy J, Pommey S (2015). The protein tyrosine phosphatase DEP-1/PTPRJ promotes breast cancer cell invasion and metastasis. Oncogene.

