# Supplementary figures and images for: Proteomic analysis distinguishes extracellular vesicles produced by cancerous versus healthy pancreatic organoids
Source: Sci Rep. 2022 Mar 3;12:3556. doi: 10.1038/s41598-022-07451-6 (PMC8894448; doi:10.1038/s41598-022-07451-6)

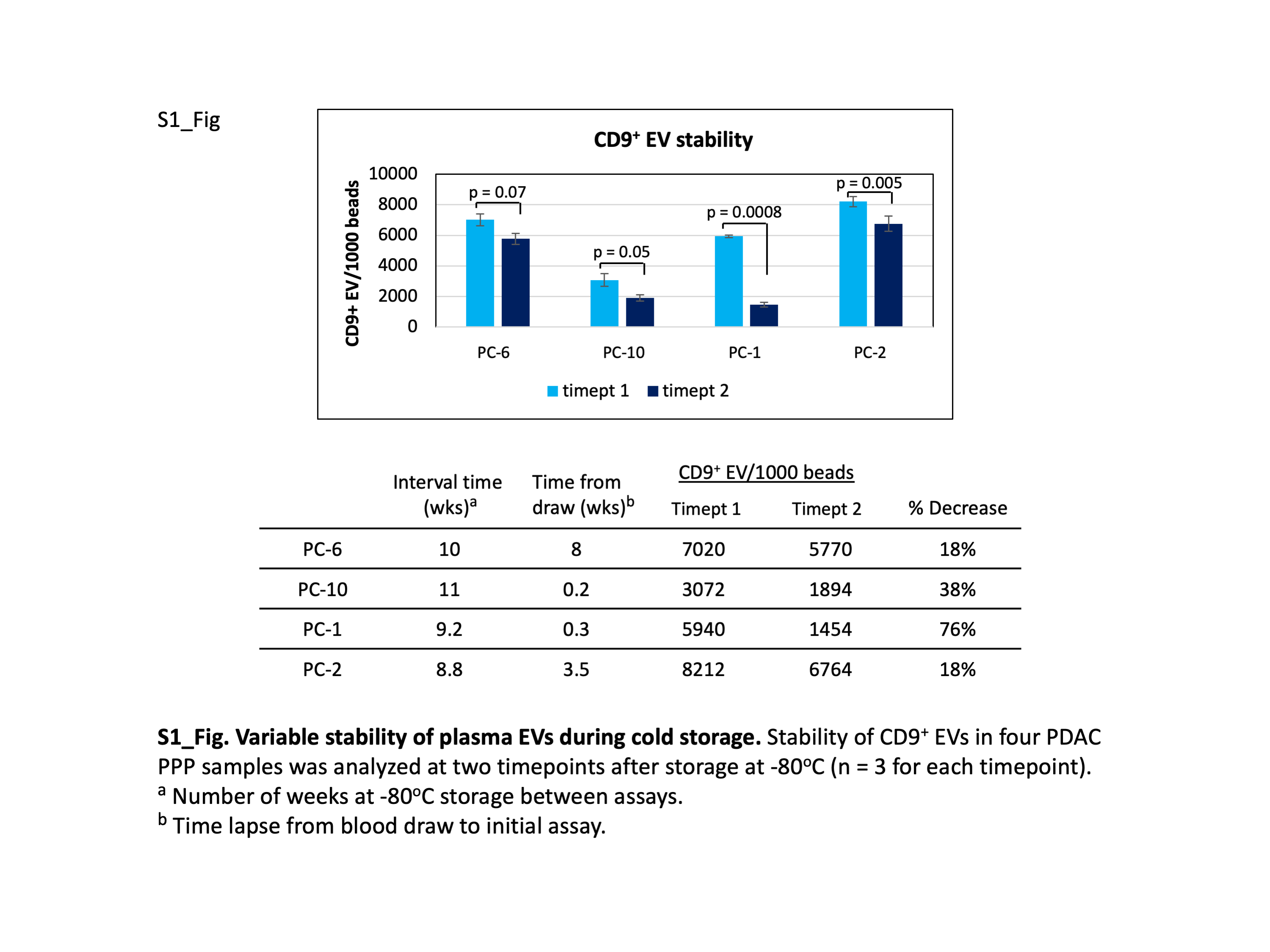

Supplement: Supplementary file 1 — Supplementary Figure S1. [file 41598_2022_7451_MOESM1_ESM.tiff]

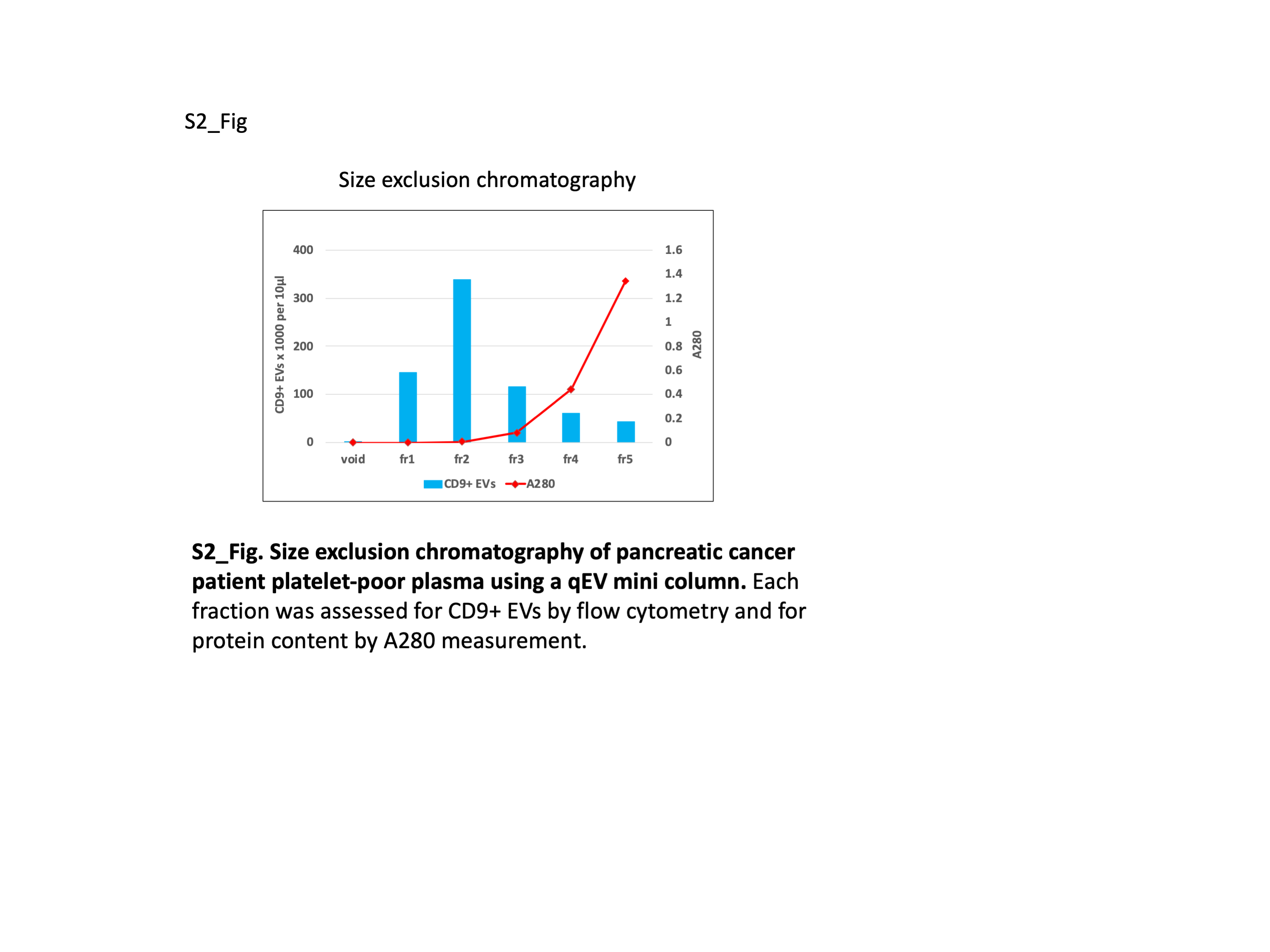

Supplement: Supplementary file 2 — Supplementary Figure S2. [file 41598_2022_7451_MOESM2_ESM.tiff]

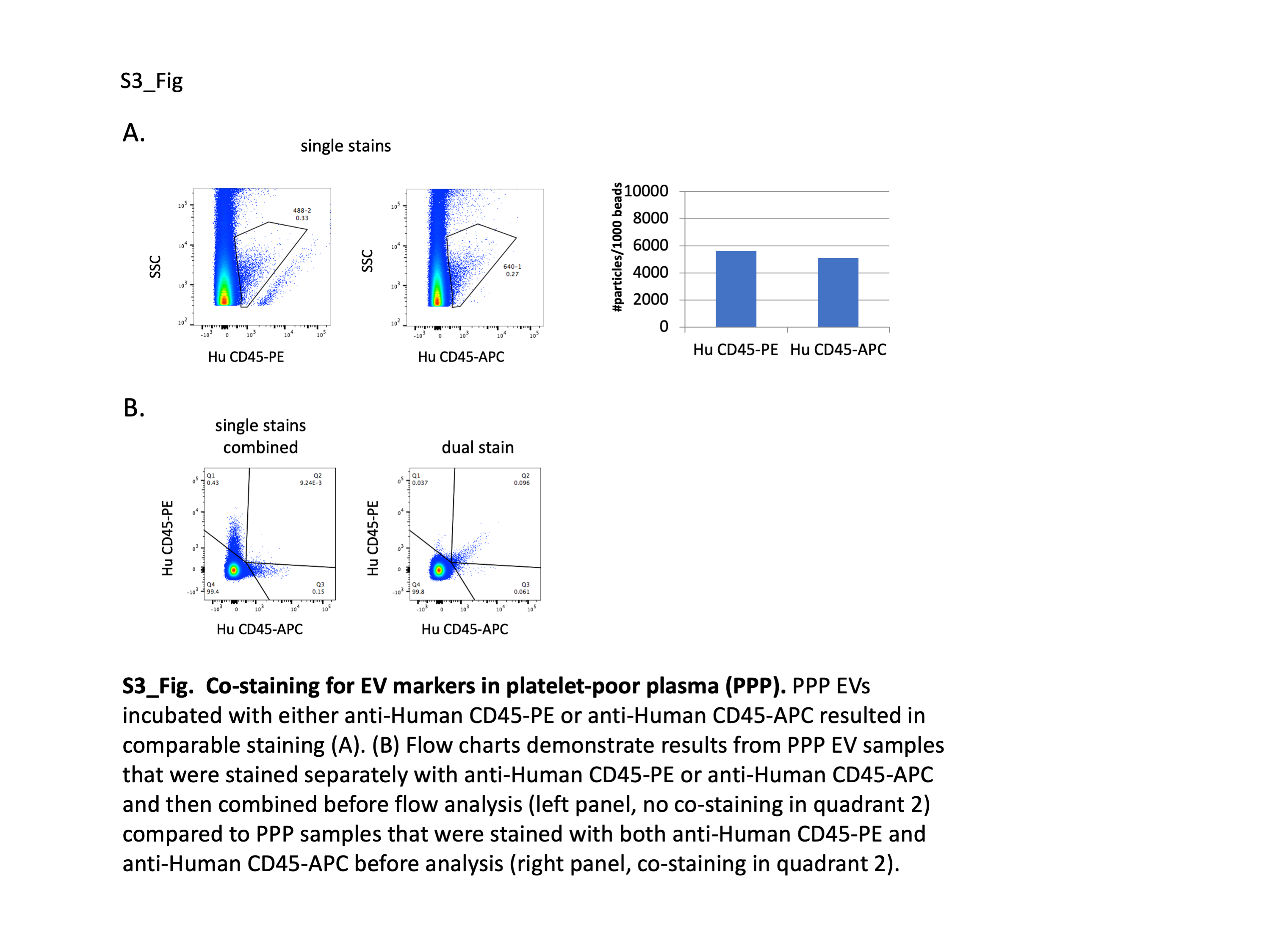

Supplement: Supplementary file 3 — Supplementary Figure S3. [file 41598_2022_7451_MOESM3_ESM.tiff]

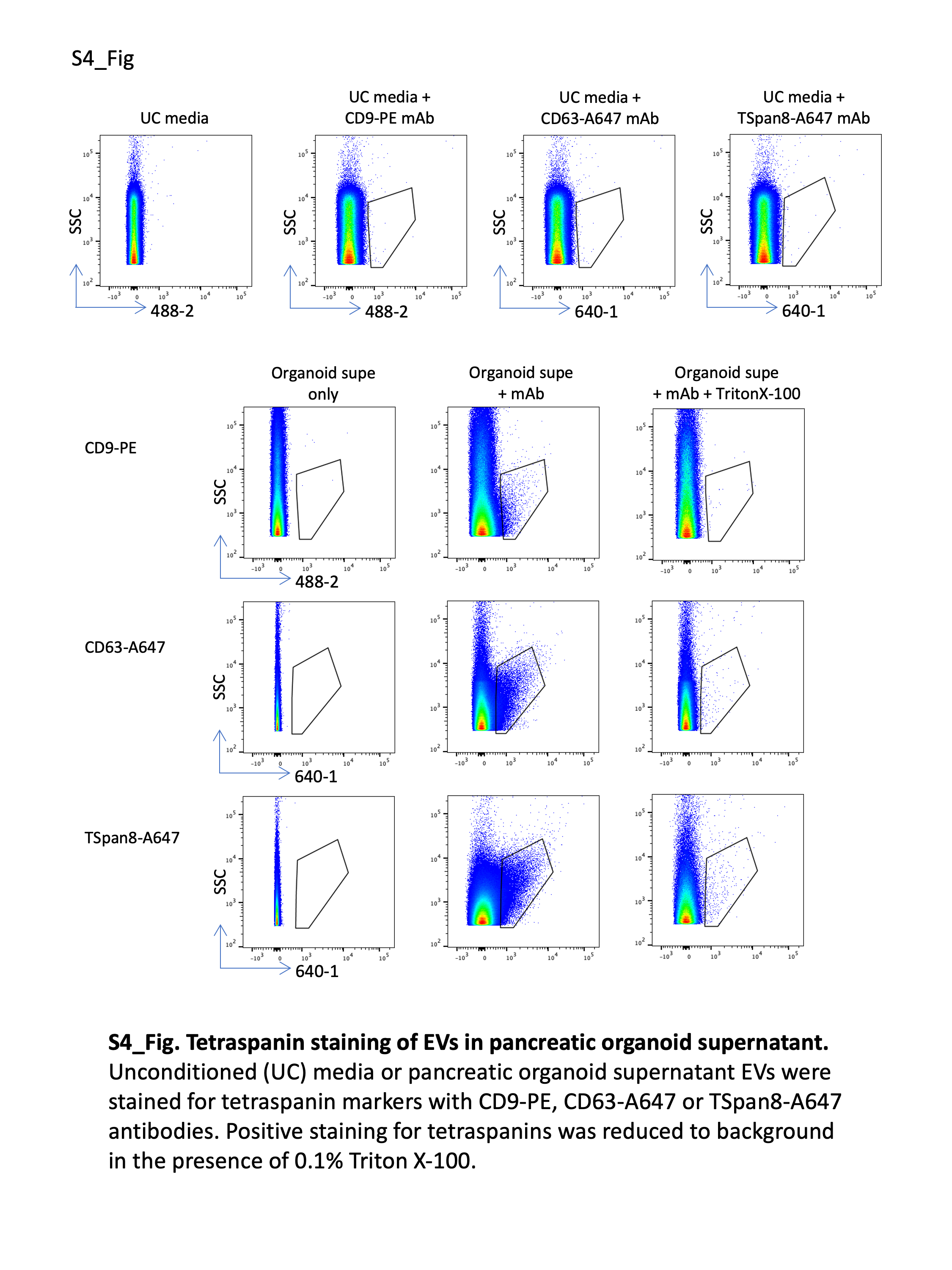

Supplement: Supplementary file 4 — Supplementary Figure S4. [file 41598_2022_7451_MOESM4_ESM.tiff]

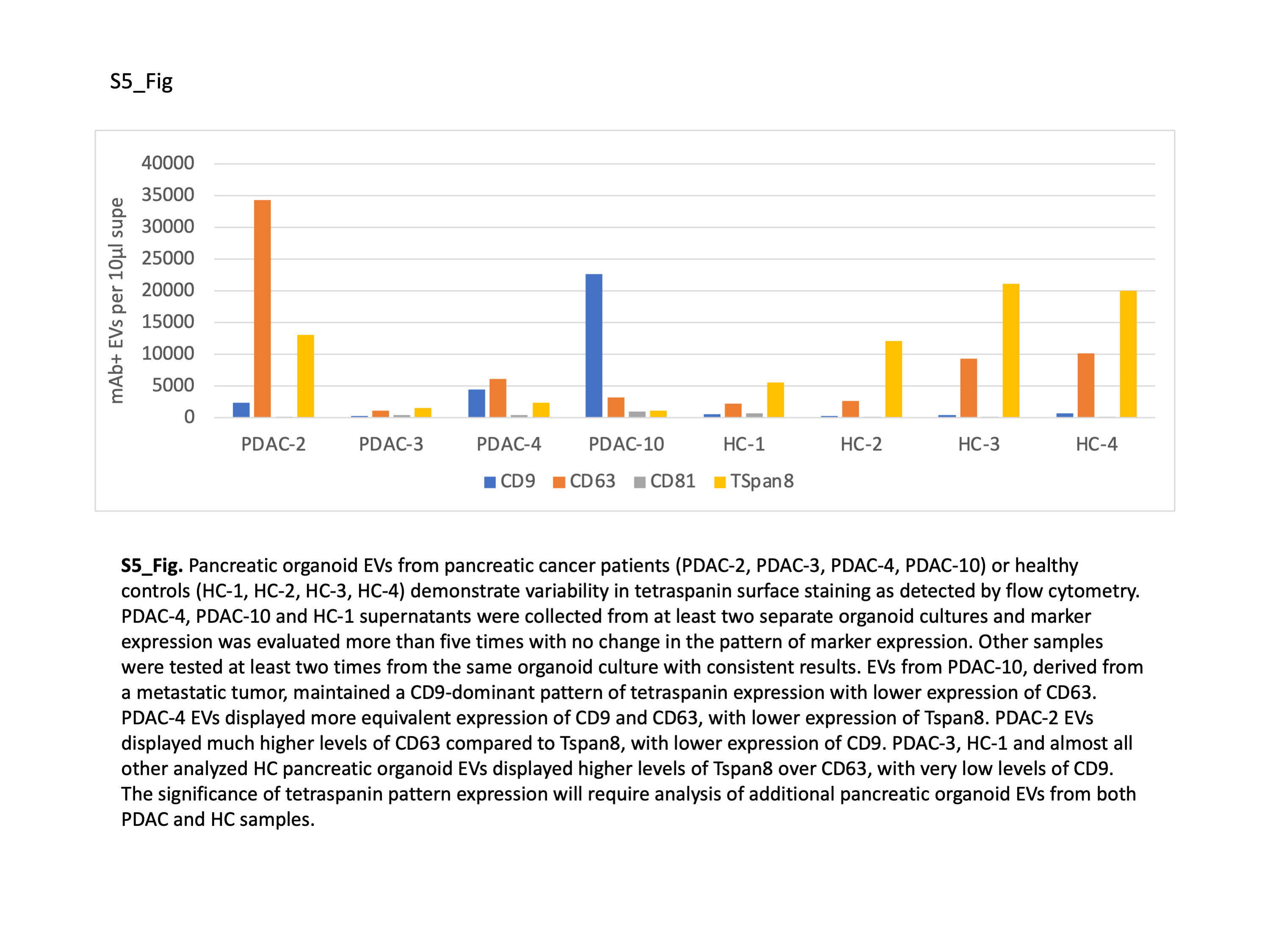

Supplement: Supplementary file 5 — Supplementary Figure S5. [file 41598_2022_7451_MOESM5_ESM.tiff]

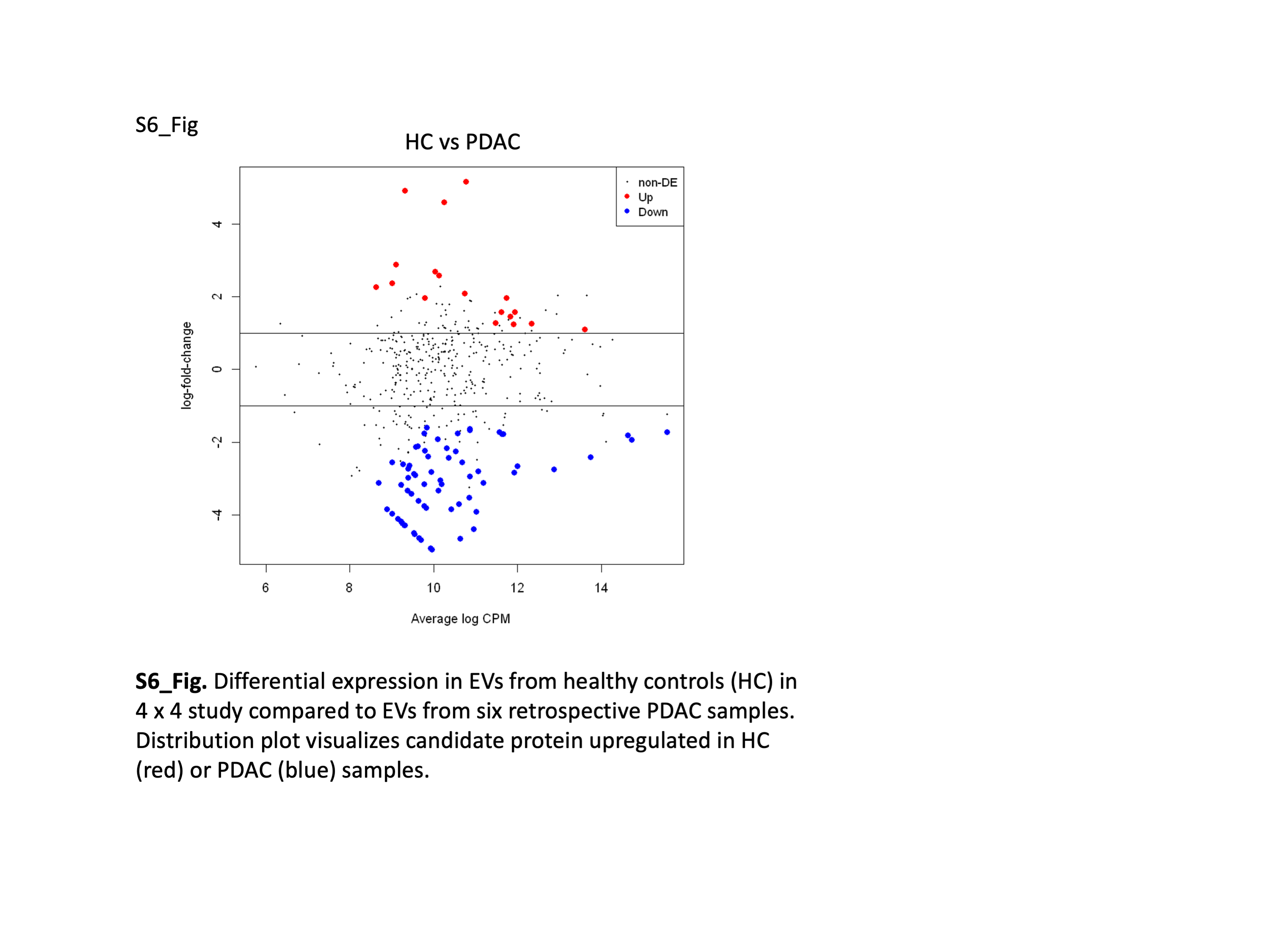

Supplement: Supplementary file 6 — Supplementary Figure S6. [file 41598_2022_7451_MOESM6_ESM.tiff]
