# Supplementary material for: Proteomic analysis distinguishes extracellular vesicles produced by cancerous versus healthy pancreatic organoids
Source: Sci Rep. 2022 Mar 3;12:3556. doi: 10.1038/s41598-022-07451-6 (PMC8894448; doi:10.1038/s41598-022-07451-6)
Supplement: Supplementary file 7 — Supplementary Table S1. [file 41598_2022_7451_MOESM7_ESM.docx]

Supplementary Table S1

Plasma samples obtained from pancreatic cancer patients for EV marker analysis.

mAb^+^ EVs per 10µl PPP^b^

______________________________

| PPP sample^a^ | CD9^+^ | CD45^+^ | CD41^+^ | Age (yrs)/ gender | Tumor size^c^ (mm) |
| --- | --- | --- | --- | --- | --- |
| No Treatment | |  |  |  |  |
| PC-1 | 5940 + 84 | 3278 + 209 | 230 + 29 | 53yo/F | 13 x 11 |
| PC-2 | 8212 + 325 | 2136 + 143 | 430 + 11 | 82yo/M | 32 x 28 |
| PC-3 | 4234 + 207 | 3000 + 65 | 808 + 2 | 74yo/F | 79 x 34 |

| Cycle 1 - 2 |  |  |  |  |  |
| --- | --- | --- | --- | --- | --- |
| PC-4 | 1593 + 76 | 1788 + 189 | 65 + 9 | 66yo/M | 56 x 37 x 32 |
| PC-5 | 4281 + 342 | 3438 + 295 | 309 + 36 | 74yo/F | 38 x 39 |
| PC-6 | 7020 + 388 | 2791 + 43 | 189 + 5 | 62yo/M | 31 x 25 |
| PC-7 | 1692 + 123 | 2639 + 126 | 13 + 1 | 82yo/F | 24 |
| PC-8 | 4372 + 162 | 7441 + 573 | 1078 + 180 | 48yo/F | None detected |

| Cycle 3 - 4 |  |  |  |  |  |
| --- | --- | --- | --- | --- | --- |
| PC-9 | 1921 + 129 | 1364 + 49 | 39 + 9 | 75yo/M | 56 x 44 |
| PC-10 | 3072 + 405 | 2813 + 264 | 266 + 23 | 54yo/M | 59 x 51 |
| PC-11 | 4456 + 329 | 2911 + 134 | 375 + 10 | 72yo /M | * |
| PC-12 | 2832 + 175 | 3129 + 128 | 37 + 3 | 72yo/M | 50 x 34 |
| PC-13 | 2257 + 58 | 4471 + 265 | 160 + 7 | 77yo/F | 15 x 12 |

^a^ Platelet-poor plasma was obtained from pancreatic cancer patients undergoing 1 to 4 cycles of neoadjuvant treatment or no treatment.

^b^ CD9, CD45 or CD41 positive EVs were detected by flow cytometry as shown in Figure 1C.

^c^ Tumor dimensions determined from patient CT scans.
