# Supplementary material for: Proteomic analysis distinguishes extracellular vesicles produced by cancerous versus healthy pancreatic organoids
Source: Sci Rep. 2022 Mar 3;12:3556. doi: 10.1038/s41598-022-07451-6 (PMC8894448; doi:10.1038/s41598-022-07451-6)
Supplement: Supplementary file 8 — Supplementary Table S2. [file 41598_2022_7451_MOESM8_ESM.docx]

Supplementary Table S2

Pancreatic tumor samples used for the generation of organoid cultures.

| Tumor | Age (yrs) | Gender | Stage | Tumor size (mm) | Tumor location |
| --- | --- | --- | --- | --- | --- |
|  |  |  |  |  |  |
| PDAC-1 | 78 | F | 1B | 27 | Pancreas |
| PDAC-2 | 67 | M | 2B | 30 | Pancreas |
| PDAC-3 | 66 | F | 2B | 28 | Pancreas |
| PDAC-4 | 70 | F | 3 | 40 | Ampulla of Vater, pancreaticobiliary |
| PDAC-5 | 80 | F | 2B | 48 | Pancreas |
| PDAC-6 | 45 | F | 2B | 27 | Pancreas |
| PDAC-7 | 49 | M | 2B | 35 | Pancreas |
| PDAC-8 | 70 | M | 2B | 28 | Pancreas |
| PDAC-9 | 64 | M | met |  | Liver |
| PDAC-10 | 52 | F | met |  | Liver |
