# Supplementary material for: Proteomic analysis distinguishes extracellular vesicles produced by cancerous versus healthy pancreatic organoids
Source: Sci Rep. 2022 Mar 3;12:3556. doi: 10.1038/s41598-022-07451-6 (PMC8894448; doi:10.1038/s41598-022-07451-6)
Supplement: Supplementary file 9 — Supplementary Table S3. [file 41598_2022_7451_MOESM9_ESM.docx]

Supplementary Table S3

Media components for pancreatic organoid cultures:

| Component | Manufacturer | Catalog# | [Working] |
| --- | --- | --- | --- |
| Advanced DMEM/F12 | Fisher/Life Tech | 12634-028 | 1x |
| HEPES (1M) | Fisher/Life Tech | 26060-CI | 10 mM |
| Glutamax (100x) | Fisher/Life Tech | 35050-061 | 1x |
| Pen/Strep/Amphotericin (100x) | Gibco | 15240062 | 1x |
| B-27 (50x) | Fisher/Gibco | 17504044 | 1x |
| N-2 (100x) | Fisher/Gibco | 17502048 | 1x |
| huEGF (50 µg/ml) | Gold Bio | 1150-04-100 | 50 ng/ml |
| FGF10 (50 µg/ml) | Peprotech | 100-26-25UG | 50 ng/ml |
| Nicotinamide (1M) | Sigma | N0636-100G | 10 mM |
| R-spondin conditioned media (10x) | N/A | N/A | 1x |
| huNoggin (100 µg/ml) | Peprotech | 120-10C-100UG | 100 ng/ml |
| huWnt3A (100 µg/ml) | Fisher/R&D Systems | 5036WN010 | 100 ng/ml |
| N-Acetyl cyteine (0.5M) | Sigma | A-7250 | 1.25 mM |
| SB431542 (12 mM) | Tocris | 1614 | 6 µM |
| Matrigel, GFR, phenol-red free | Fisher/Corning | CB-40230C | 1x |
