# Supplementary material for: Proteomic analysis distinguishes extracellular vesicles produced by cancerous versus healthy pancreatic organoids
Source: Sci Rep. 2022 Mar 3;12:3556. doi: 10.1038/s41598-022-07451-6 (PMC8894448; doi:10.1038/s41598-022-07451-6)
Supplement: Supplementary file 10 — Supplementary Table S4. [file 41598_2022_7451_MOESM10_ESM.docx]

Supplementary Table S4: MIFlowCyt-EV Worksheet

| **Framework Criteria** |  |
| --- | --- |
| 1.1 Preanalytical variables conforming to MISEV guidelines. | **Plasma EVs:** Blood samples were collected into vacutainer tubes containing EDTA to prevent coagulation and all samples were processed within two hours of the draw. Samples were centrifuged twice at 2500 x g for 15 mins at room temperature to obtain platelet-poor plasma (PPP) and supernatant was transferred into clean tubes after each spin, taking care to avoid contamination from the blood cell pellet. PPP was then aliquoted and stored at -80^o^C. Samples were thawed only once before analysis. Unless specified otherwise, all plasma samples in this study were stained and analyzed by flow cytometry within one week of processing. **Organoid EVs:** Supernatant was collected and replaced with fresh media every two days and stored at -80C after sequential centrifugation at 300 x g for 10 minutes and at 2000 x g for 10 minutes, both at room temperature. Clarified supernatants were stored at -80^o^C prior to screening or isolation. |
| 1.2 Experimental design according to MIFlowCyt guidelines. | **1.1 Aim:** Platelet-poor plasma samples were obtained from pancreatic cancer patients undergoing neoadjuvant treatment for preliminary assessment of differences in EV marker levels. As EV marker detection in plasma samples provided poor designation of tissue source, organoid cultures from pancreatic cancer and healthy tissue were generated as a more defined source of EVs. EVs isolated from organoid supernatants were analyzed by mass spectrometry to compare profiles of highly expressed proteins in pancreatic cancer versus healthy controls. **1.2 Keywords:** Pancreatic ductal adenocarcinoma, PDAC, extracellular vesicles, high resolution flow cytometry, mass spectrometry, EV markers, biomarkers; **1.3 Experimental variables:** Plasma EV samples from 13 pancreatic cancer patients were assessed by fluorescence-triggered flow cytometry staining for CD9, CD45 and CD41. Pancreatic organoid supernatant EVs were obtained for 10 pancreatic cancer patients and 4 healthy controls, screened for tetraspanin expression by fluorescence-triggered flow cytometry and isolated by size exclusion chromatography for proteomic analysis. |
| 2.1 Sample staining details | 10µl of PPP sample, clarified supernatant or SEC column fraction was incubated with 1µl of labeled monoclonal antibody in 0.65ml polypropylene microfuge tubes. Samples were stained at a predetermined optimal concentration of antibody for 2hrs at room temperature, protected from light. |
| 2.2 Sample washing details | No washing steps were performed. |
| 2.3 Sample dilution details | All dilutions of sample and reagents were made with 0.1µm-filtered PBS.  **Plasma EVs:** Nanoparticle density (as detected by flow cytometry) was highly variable in patient PPP, so final dilution of stained samples was determined empirically such that the event rate did not exceed 10K events per second. 10µl of a freshly diluted 200nm bead standard (Fluoresbrite YG Microsphere beads) was added as an internal reference to the stained EV samples prior to dilution, and acquisition of data stopped after a set number (1000) of beads were collected. **Organoid supernatant EVs:** Stained samples were diluted to 1:25 and data acquired for 3 minutes under the medium flow rate. |
| 3.1 Buffer alone controls. | For all samples, PBS (0.1µm-filtered) was run through the cytometer before each use until background event rates dropped below 100 events/second. PBS samples were then acquired for 1 minute and recorded as the background rate for each run. All settings, including threshold, voltage and flow rate, were the same as those used during sample acquisition. |
| 3.2 Buffer with reagent controls. | Prior to each use, antibodies were centrifuged at 12K x g for 15 minutes to remove debris and aggregates. For each monoclonal antibody used, antibody controls (PBS plus antibody alone) were run to assess for any background fluorescence signal. All settings, including threshold, voltage and flow rate, matched those used during sample acquisition. Some commercial labeled antibodies were found to have significant nanoparticle fluorescence and were excluded from our investigation. |
| 3.3 Unstained controls. | Unstained control samples were run under the same settings as stained samples and showed no fluorescence signal. |
| 3.4 Isotype controls. | Isotype controls were not consistently run with each experiment. However, non-specific fluorescent binding due to Ig isotype or dye-specific interactions did not seem to be an issue as our panel for EV marker screening included antibodies with matching isotype and dye. All settings were consistent for each sample acquisition. |
| 3.5 Single-stained controls. | Single stained controls were not normally run. On the occasion when they were run, fluorescent signal appeared only in the appropriate channel. All settings were consistent for each sample acquisition. |
| 3.6 Procedural controls. | Procedural controls for size exclusion chromatography isolation of EVs were run, but not for each experiment. No fluorescence signal was detected in SEC fractions with media or buffer. |
| 3.7 Serial dilutions. | For plasma samples with a high event rate (>10K evts/sec), serial dilutions were prepared and dilutions showing a linear decrease in event rate were used. Serial dilutions were not performed on supernatants from organoid cultures. |
| 3.8. Detergent treated EV-samples | Samples with positive fluorescence staining were treated with Triton X-100 at a final concentration of 0.1% for 5 minutes at room temperature. Acquisition of the detergent-treated samples showed >95% loss of fluorescent signal. |
| 4.1 Trigger Channel(s) and Threshold(s). | Threshold was set at 300 on SSC. FACSCanto II fluorescent trigger channels used: 488-1, 488-2, 640-1, 640-2 |
| 4.2 Flow Rate / Volumetric quantification. | Flow rate was not directly quantified. |
| 4.3 Fluorescence Calibration. | Fluorescence calibration was not implemented. |
| 4.4 Light Scatter Calibration. | Light scatter calibration was not implemented. |
| 5.1 EV diameter/surface area/volume approximation. | EV diameter, surface area, and volume were not determined. |
| 5.2 EV refractive index approximation. | EV refractive index was not determined. |
| 5.3 EV epitope number approximation. | EV epitope number was not determined. |
| 6.1 Completion of MIFlowCyt checklist. | Completed |
| 6.2 Calibrated channel detection range | Fluorescence and light scatter calibration was not implemented. |
| 6.3 EV number/concentration. | Antibody-positive EV numbers per volume was reported for plasma samples and some SEC fractions. |
| 6.4 EV brightness. | EV brightness was not determined. |
| 7.1. Sharing of data to a public repository. | Further details of flow cytometry settings or results may be obtained by contacting the corresponding author. |
