# Supplementary material for: Proteomic analysis distinguishes extracellular vesicles produced by cancerous versus healthy pancreatic organoids
Source: Sci Rep. 2022 Mar 3;12:3556. doi: 10.1038/s41598-022-07451-6 (PMC8894448; doi:10.1038/s41598-022-07451-6)
Supplement: Supplementary file 11 — Supplementary Table S5. [file 41598_2022_7451_MOESM11_ESM.doc]

Supplementary Table S5: MIFlowCyt Worksheet

| **Requirement** | **Requested Information** |
| --- | --- |
| 1.1. Purpose | Platelet-poor plasma samples were obtained from pancreatic cancer patients undergoing neoadjuvant treatment for preliminary assessment of differences in EV marker levels. As EV marker detection in plasma samples provided poor designation of tissue source, organoid cultures from pancreatic cancer and healthy tissue were generated as a more defined source of EVs. EVs isolated from organoid supernatants were analyzed by mass spectrometry to compare profiles of highly expressed proteins in pancreatic cancer versus healthy controls. |
| 1.2. Keywords | Pancreatic ductal adenocarcinoma, PDAC, extracellular vesicles, high resolution flow cytometry, mass spectrometry, EV markers, biomarkers |
| 1.3. Experiment variables | Plasma EV samples from 13 pancreatic cancer patients were assessed by fluorescence-triggered flow cytometry staining for CD9, CD45 and CD41. Pancreatic organoid supernatant EVs were obtained for 10 pancreatic cancer patients and 4 healthy controls, screened for tetraspanin expression by fluorescence-triggered flow cytometry and isolated by size exclusion chromatography for proteomic analysis. |
| 1.4. Organization name and address | Oregon Health & Science University; 3181 SW Sam Jackson Park Road, Portland, Oregon 97239 |
| 1.5. Primary contact name and email address | Abby Buenafe, Ph.D.; buenafea@ohsu.edu |
| 1.6. Date or time period of experiment | 2017 - 2021 |
| 1.7. Conclusions | EV markers that Identify tissue source are needed in the analysis of human plasma EVs. Flow cytometric detection of EVs at the single particle level can be used to screen for EV markers in supernatant and size exclusion chromatography fractions. |
| 1.8. Quality control measures | Standardized collection, processing and storage of samples. Standardized sample volume and timing for antibody staining. Fluorescent beads with known size range for cytometer check. Standardized acquisition settings. |
| 2.1.1.1. (2.1.2.1., 2.1.3.1.) Sample description | Platelet-poor plasma; clarified supernatants; size-exclusion chromatography fractions |
| 2.1.1.2. Biological sample source description | Platelet-poor plasma samples were obtained from pancreatic cancer patients. Organoid cultures from healthy pancreas and pancreatic cancer tissue were generated as a more defined source of supernatant EVs. |
| 2.1.1.3. Biological sample source organism description | human |
| 2.1.2.2. Environmental sample location | na |
| 2.3. Sample treatment description | untreated |
| 2.4. Fluorescence reagent(s) description | 10µl of PPP sample, clarified supernatant or SEC column fraction was incubated with 1µl of labeled monoclonal antibody in 0.65ml polypropylene microfuge tubes. Samples were stained at a predetermined optimal concentration of antibody for 2hrs at room temperature, protected from light. |
| 3.1. Instrument manufacturer | Becton-Dickinson |
| 3.2. Instrument model | FACSCanto II with adjustable laser modification |
| 3.3. Instrument configuration and settings | Threshold: SSC, 300  FSC: 475V; SSC: 450V; 488-1 (535/30): 650V; 488-2 (585/42): 604V; 640-1 (670/30): 750V; 640-2 (780/60): 650V  Lasers: 375nm, 16mW; 488nm, 200 mW; 640nm, 75mW. |
| 4.1. List-mode data files | Further details of flow cytometry settings or results may be obtained by contacting the corresponding author. |
| 4.2. Compensation description | none |
| 4.3. Data transformation details | none |
| 4.4.1. Gate description | Exclusion of signal from unlabeled sample and antibody-only controls |
| 4.4.2. Gate statistics | none |
| 4.4.3. Gate boundaries | 100 – 300nm; fluorescence-triggered |
