# Supplementary material for: Proteomic analysis distinguishes extracellular vesicles produced by cancerous versus healthy pancreatic organoids
Source: Sci Rep. 2022 Mar 3;12:3556. doi: 10.1038/s41598-022-07451-6 (PMC8894448; doi:10.1038/s41598-022-07451-6)
Supplement: Supplementary file 12 — Supplementary Table S6. [file 41598_2022_7451_MOESM12_ESM.docx]

Supplementary Table S6: Flow cytometry reagents
