# Supplementary material for: Proteomic analysis distinguishes extracellular vesicles produced by cancerous versus healthy pancreatic organoids
Source: Sci Rep. 2022 Mar 3;12:3556. doi: 10.1038/s41598-022-07451-6 (PMC8894448; doi:10.1038/s41598-022-07451-6)
Supplement: Supplementary file 13 — Supplementary Table S7. [file 41598_2022_7451_MOESM13_ESM.docx]

**Supplementary Table S7**

**Normalized mass spectrometry spectral counts from pancreatic organoid EVs in the 4 x 4 study.**

^1^ Protein accession ID.

^2^ EV samples derived from pancreatic cancer (PDAC) or healthy control (HC) organoids supernatants after size exclusion chromatography. Numbers represent total spectral count. PDAC stage: PDAC-1 (1B); PDAC-2 (2B); PDAC-3 (2B); PDAC-4 (3).

^3^ Average spectral count of four PDAC EV samples. ^4^ Average spectral count of four HC EV samples.

^5^ PValue is the p-value derived from the EdgeR test

^6^ FDR is the p-value after a Benjamini–Hochberg multiple-testing correction is applied to limit the false-discovery rate.
