# Supplementary material for: Proteomic analysis distinguishes extracellular vesicles produced by cancerous versus healthy pancreatic organoids
Source: Sci Rep. 2022 Mar 3;12:3556. doi: 10.1038/s41598-022-07451-6 (PMC8894448; doi:10.1038/s41598-022-07451-6)
Supplement: Supplementary file 14 — Supplementary Table S8. [file 41598_2022_7451_MOESM14_ESM.docx]

**Supplementary Table S8**

**GO annotations for 37 proteins upregulated in healthy control pancreatic organoid EVs.**
