# Supplementary material for: Proteomic analysis distinguishes extracellular vesicles produced by cancerous versus healthy pancreatic organoids
Source: Sci Rep. 2022 Mar 3;12:3556. doi: 10.1038/s41598-022-07451-6 (PMC8894448; doi:10.1038/s41598-022-07451-6)
Supplement: Supplementary file 15 — Supplementary Table S9. [file 41598_2022_7451_MOESM15_ESM.docx]

**Supplementary Table S9**

**GO annotations for 31 proteins upregulated in PDAC pancreatic organoid EVs.**
